# Carboxylic Acid-Cured Epoxidised Soybean Oil under Catalyst-Free Conditions: Kinetics and Sustainable Packaging Potential

**DOI:** 10.1021/acsomega.5c11969

**Published:** 2026-08-18

**Authors:** Ana Barros, Neymara Nepomuceno, Andreas Ries, Carlos Luna, Marcus Fook, Edcleide Araújo, Renate Wellen

**Affiliations:** † Academic Unit of Materials Engineering, 154624 Federal University of Campina Grande, Av. Aprígio Veloso, 882 - Bodocongó, 58429-900 Campina Grande, Paraíba, Brazil; ‡ Polytechnic Faculty, National University of Asunción, 111421 San Lorenzo, Paraguay; § Department of Materials Engineering, Federal University of Paraíba, Cidade Universitária, 58051-900 João Pessoa, PB, Brazil

## Abstract

This work investigates the curing behavior of epoxidised soybean oil (ESO) with citric (CA), maleic (MA), and salicylic (SA) acids. The curing process was monitored by Fourier-transform infrared (FTIR) and dynamic differential scanning calorimetry (DSC) at heating rates of 5–20 °C min^–1^. FTIR analysis showed a progressive decrease of the epoxide band (∼950 cm^–1^) for ESO/CA and ESO/SA systems, while ESO/MA retained oxirane signals after thermal treatment, indicating incomplete curing. ESO/CA and ESO/SA exhibited gel contents above 80% and low swelling, with cross-link densities of ∼1.7–2.1 mmol cm^–3^, whereas ESO/MA presented a much lower value (∼0.3 mmol cm^–3^). DSC analysis revealed two exothermic curing stages for ESO/CA and ESO/SA that shifted to higher temperatures, consistent with primary cross-linking followed by secondary reactions. Cure kinetics were evaluated using the Friedman, Vyazovkin, Ozawa–Flynn–Wall (OFW), and Kissinger–Akahira–Sunose (KAS) models. Model-free Friedman and Vyazovkin approaches provided the best description of the experimental data (*R* ^2^ > 0.99), revealing conversion-dependent activation energies that increased with the degree of conversion, reaching ∼85 kJ mol^–1^ for CA and ∼87 kJ mol^–1^ for SA systems. In contrast, OFW and KAS models showed lower fitting quality (*R* ^2^ < 0.88), indicating that single-activation-energy approaches are insufficient to describe the curing process. Tensile tests confirmed structure–property differences between the networks: ESO/CA exhibited higher modulus and strength (∼0.85 and ∼0.55 MPa), while ESO/SA showed greater elongation (∼147%). Overall, catalyst-free ESO/CA and ESO/SA systems form cross-linked biobased thermosets with tunable mechanical behavior and well-defined curing kinetics, demonstrating their potential for the development of sustainable epoxy materials.

## Graphical abstract

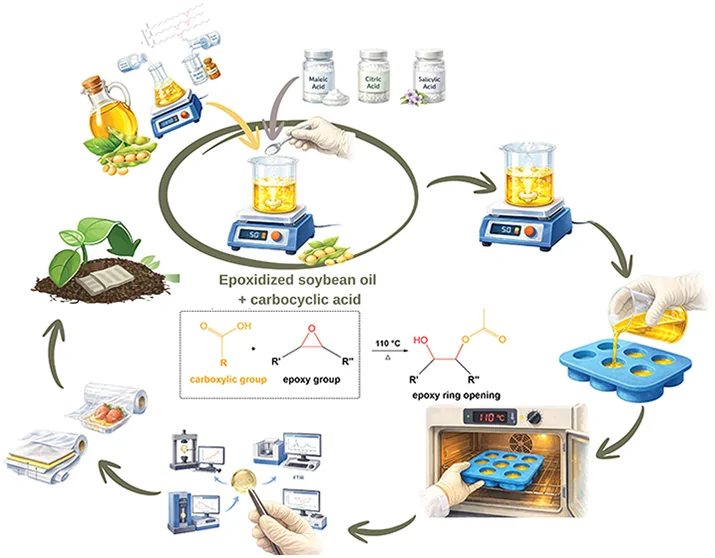

## Introduction

Epoxy resins are widely used in various industrial sectors due to their exceptional mechanical, thermal, and adhesion properties. However, conventional production of these resins relies significantly on fossil sources, raising environmental concerns, particularly linked to greenhouse gas emissions and dependence on nonrenewable resources.^1^ In the context of epoxy resins traditionally derived from petrochemical precursors, there is a growing interest in biobased alternatives that can offer comparable performance, combined with a smaller environmental footprint.^2,3^

Epoxidized vegetable oils have been widely investigated as sustainable monomers for epoxy systems due to their renewable character, availability, and potential to replace petrochemical-based materials. Among them, soybean oil stands out because of its large global production, relatively low cost, and broad industrial availability.^4^ In addition to its use in the food industry, soybean oil has also been employed in technological applications such as lubricants during PVC processing.^5^ Its chemical structure is particularly attractive for epoxy resin synthesis, as the presence of unsaturated bonds enables straightforward epoxidation and further tailoring of material properties according to specific applications.^6^ Although soybean cultivation may be associated with environmental concerns such as land-use change and biodiversity impacts when inadequately managed, the valorization of established agro-industrial production streams, especially those linked to the food industry, may contribute to more sustainable material development without necessarily increasing land demand.

In this context, epoxidized soybean oil (ESO) has proven to be one of the most promising alternatives to produce biobased epoxy resins.^7,8^ ESO is obtained by epoxidizing the triglycerides contained in soybean oil, in which epoxy groups are inserted into the unsaturated lipid chains, making it reactive and suitable for use as a matrix in epoxy resin systems.^9^ They exhibit attractive features such as a high degree of functionality (double bonds, epoxy functions, and ester groups in the same molecular structure), relatively low cost, making them promising candidates to generate fully biobased thermosets with inherent reprocessability and recyclability.^10–12^ However, their oxirane groups in the chain exhibit lower reactivity, resulting in low (glass transition temperature) Tg and poor mechanical properties compared to petrochemical monomers bearing epoxy glycidyl functions.^13–15^

The curing process of epoxy resins is a determining factor for their final properties. It influences the rigidity and mechanical resistance as well as the thermal and chemical stability of the final material. During the curing of epoxy resins, the epoxy groups present in the polymer matrix are cross-linked with curing agents and form a three-dimensional network that gives the material its characteristic properties.^16,17^ For biobased epoxy resins, the choice of curing agent is crucial as it has a direct impact on the curing kinetics and consequently on the performance of the final material.

Carboxylic acids (CAR) are widely available and economically viable tricarboxylic acids. They are known for their ability to form dense polymer networks, which can result in materials with high rigidity and thermal resistance. Furthermore, their multifunctional nature can promote higher cross-linking density, which is desirable in many industrial applications.^18,19^ From a broad spectrum of possibilities, citric acid (CA), maleic acid (MA), and salicylic acid (SA) have been widely investigated as curing agents for biobased epoxy resins. Citric acid and maleic acid have been extensively studied due to their multifunctional carboxylic structures and availability,^7,8,18,19^ while salicylic acid has attracted attention for its aromatic structure and potential functional properties in polymer networks.^20,21^

These acids can influence the cross-linking process forming esters with alcohols. Their acidic property enables participation in neutralization reactions with bases, which can catalyze or modulate the curing rate in epoxy systems. Polarity affects the uniformity of the polymer network formed during curing, directly impacting the mechanical and thermal properties of the final material. Additionally, the ability to form hydrogen bonds enhances the cohesion of the polymer network, improving the thermal and mechanical stability of the cured material. Lastly, unsaturation in the chain can engage in further reactions, such as addition, polymerization or Diels–Alder reactions, providing additional cross-linking points or modulating the rigidity of the polymer.^16,17^ In an ideal formulation, the melting temperature of the acid should be below the curing temperature, to ensure the complete dissolution of the acid groups in the epoxy monomer before the onset of curing reactions, which usually occurs between 120–150 °C.^21^

Moser prepared fully biobased epoxy resins by curing ESO with itaconic acid and citric acid, showing that the higher functionality of citric acid compared to itaconic acid resulted in increased Tg, thermal stability, tensile strength, and storage modulus.^19^ Maleic acid (MA), with its dicarboxylic structure and one C=C double bond, can introduce rigidity into the polymer network and modulate the curing rate of the resin. Its distinct reactivity may influence both the glass transition temperature and the mechanical properties of the resulting material. The presence of unsaturations and carboxylic groups in MA has been reported to increase the rigidity and Tg of epoxy networks, leading to improved thermal and mechanical resistance compared to more flexible systems lacking such functionalities.^22,23^

Despite these promising attributes, the reactivity of maleic acid toward epoxidized vegetable oils can be limited under catalyst-free curing conditions. The rigid and planar molecular structure of MA, combined with its cis-dicarboxylic configuration, may restrict molecular mobility during network formation and favor the formation of monoester structures, leaving a fraction of epoxy groups unreacted. Moreover, the acid–epoxy reaction proceeds relatively slowly in the absence of catalysts and at moderate curing temperatures, and diffusion limitations arising after gelation can further hinder complete epoxy conversion. These effects may be exacerbated by solubility and processing constraints, as maleic acid requires predissolution in polar solvents to ensure homogenization with the epoxy monomer. Consequently, ESO systems cured with MA may exhibit residual oxirane functionalities and reduce cross-linking efficiency compared to systems cured with more flexible or highly functional carboxylic acids, such as citric acid, under similar curing conditions.

Salicylic acid (SA), containing an aromatic ring and a hydroxyl group, can impart additional rigidity to the polymer network, resulting in materials with enhanced thermal and mechanical properties, particularly in terms of heat resistance and durability.^8,24,25^ Furthermore, SA is a safe compound widely used in the pharmaceutical industry due to its antibacterial, anti-infectious, and antifungal activities.^26^ SA has been extensively investigated for applications in food packaging, with the primary objective of extending the shelf life of fruits by protecting them against the activity of microorganisms such as fungi and bacteria.^27,28^

Importantly, ESO-based epoxy networks derived from renewable resources have attracted increasing attention as potential sustainable alternatives to petrochemical thermosets. The use of naturally occurring carboxylic acids such as citric and salicylic acids further contributes to the development of biobased polymer systems. These materials may offer interesting opportunities for coatings and packaging-related applications due to their cross-linked structure and tunable mechanical properties. However, the suitability of such systems for food-contact applications requires additional investigations, including permeability measurements, migration studies, and long-term stability assessments under realistic service conditions. However, despite the growing interest in ESO-based thermosets, the curing kinetics and network formation mechanisms of these systems remain insufficiently understood.

Moreover, the analysis of curing kinetics is essential to understand how the nature of the curing agent affects cross-linking reactions and to optimize processing conditions for epoxy systems.^29^ Differential scanning calorimetry (DSC) is widely employed for this purpose, enabling the determination of key parameters such as activation energy, degree of conversion, and glass transition temperature.^30,31^ However, many studies remain limited to experimental characterization, without a detailed kinetic interpretation capable of capturing the complexity of biobased epoxy curing reactions.

A previous study by our research group investigated epoxy thermosets derived from epoxidized soybean oil (ESO) cured with citric, maleic, and salicylic acids, focusing primarily on the thermal degradation behavior of the cured materials. In that work, thermogravimetric analysis (TG), TG-IR, and isoconversional kinetic models were applied to evaluate thermal stability and degradation kinetics of already formed networks. However, the curing process itself and the mechanisms governing network formation were not investigated in detail. In particular, aspects such as the evolution of epoxy group conversion during curing, the kinetics of the curing reaction, and the relationship between curing chemistry and the final mechanical performance of the materials remained unexplored.

Therefore, the present study focuses specifically on the curing stage of ESO-based thermosets, combining Fourier-transform infrared (FTIR) monitoring of epoxy group consumption, quantitative conversion analysis based on the Beer–Lambert law, differential scanning calorimetry (DSC) curing analysis, model-free kinetic approaches, and mechanical characterization. This approach allows a deeper understanding of the curing kinetics, reaction mechanisms, and structure–property relationships of ESO thermosets prepared with different carboxylic acids.

Considering the increasing demand for sustainable and biobased materials, ESO-based thermosets derived from renewable resources may offer interesting opportunities for coatings and packaging-related applications. However, a detailed understanding of their curing kinetics and network formation is essential before evaluating their potential suitability for such applications.

In this context, the present work investigates the curing kinetics of biobased epoxy resins based on epoxidized soybean oil (ESO) cured with citric (CA), maleic (MA), and salicylic (SA) acids, addressing the limited understanding of catalyst-free ESO–carboxylic acid systems. Dynamic DSC scans were analyzed using Friedman, Kissinger–Akahira–Sunose, Ozawa–Flynn–Wall, and Vyazovkin approaches, allowing a critical comparison of their applicability to complex biobased epoxy networks and the determination of activation energy (*E*_a_) as a function of conversion (α). By correlating curing efficiency, cross-link density, and chemical structure with functional requirements relevant to food-packaging applications, this study establishes structure–reactivity relationships that support rational process optimization and application-oriented material design. These findings position ESO-based thermosets as promising candidates to replace petrochemical-based coatings, adhesives, and structural components in the food industry.

## Methodology

### Materials

To produce the epoxy resins, ESO was used and kindly provided by BBC Química (São Paulo, Brazil), with an average epoxy equivalent weight (EEW) of 230 g/mol or 4.3 epoxy groups per molecule. EEW is the molar mass that provides 1 mol epoxy groups. Carboxylic acids, i.e., salicylic, maleic, and citric, all with a purity of 98%, were used as curing agents and were acquired from Sigma-Aldrich (São Paulo, Brazil). All reagents were used as received without further purification.

The selection of citric and salicylic acids as curing agents also considered their regulatory status for food-contact applications. Citric acid is recognized as GRAS (Generally Recognized as Safe) by the U.S. FDA, enabling direct food-contact use, while salicylic acid, though requiring controlled application, has documented antimicrobial effects desirable for active packaging. These choices allow the study to address both performance and compliance with food packaging safety standards.

### Synthesis of Epoxy Resins (ESO/CAR)

Carboxylic acids were added to ESO in a stoichiometric ratio of *R* = 1.2 epoxy/acid groups (or *R* = 0.8 acid/epoxy), which was selected based on previous studies involving curing systems of epoxidized vegetable oils with multifunctional carboxylic acids.^8,9,26^ In such systems, a slight excess of epoxy groups is commonly adopted to promote efficient network formation and minimize the presence of residual acidic species in the final material.

The same stoichiometric ratio was maintained for the ESO/MA system to ensure consistency among the studied formulations, allowing a direct comparison of the curing behavior and properties of the thermosets prepared with different carboxylic acids.

ESO was heated to 80 °C, then CAR was added, and the compounds were magnetically stirred for 10 min until reaching complete homogenization. The uncured mixtures were poured into a silicone mold and cured at 110 °C for 2 h. Since maleic and citric acids are not soluble in oil, they were predissolved in isopropyl alcohol (approximately 3 mL for each gram of acid). The procedure used is illustrated in Figure 1 and Table S1 (Supporting Information) presents the produced compositions with their respective amounts and codifications.

**Figure 1:**
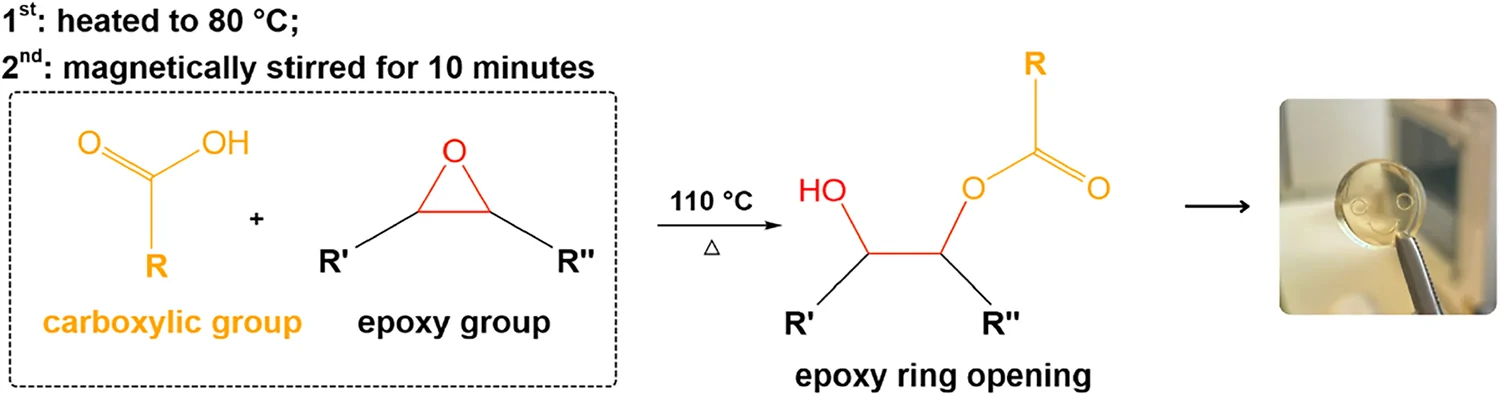
Scheme of processing the epoxy systems based on ESO and CAR.

Isopropyl alcohol was used solely as a temporary processing aid to dissolve carboxylic acids with limited solubility in ESO, enabling homogeneous mixing prior to curing. Nevertheless, for food-contact applications, residual solvent content must be assessed, and future studies will include solvent removal validation and migration testing to ensure compliance with regulatory limits.

All synthesis steps were conducted to minimize potential contamination relevant to subsequent food-contact testing, including the use of food-grade solvents where possible and curing profiles designed to reduce unreacted monomers. These considerations ensure that, in future migration and compliance tests, the formulations will have lower residual reactivity and reduced potential for extractables.

### Fourier Transform Infrared Spectroscopy (FTIR)

Spectra were collected in the wavenumber range of 4000–650 cm^–1^, with 16 scans and a 4 cm^–1^ resolution, using the Attenuated Total Reflection (ATR) mode at room temperature. The equipment used was a PerkinElmer Spectrum 400 (Waltham, Massachusetts, USA), and data modeling was performed using Origin software.

The degree of cure (dα) was determined based on an adaptation of the Beer–Lambert Law,^33^ as shown in eq 1, where the reference band used (*A*_*R*_)_0→*t*_ remains constant during the whole curing - the 2853 cm^–1^ peak for ESO/CA, the 1450 cm^–1^ peak for ESO/MA, and the 1615 cm^–1^ peak for ESO/SA. The band (*A*_*C*_)_0→*t*_ corresponds to the reactive functional group that reacts during curing, in the present work the selected band was 862 cm^–1^ to 950 cm^–1^, associated with the epoxide group, whose intensity decreases throughout curing.

1 $$d\alpha =1-\frac{{\left(\frac{A_{C}}{A_{R}}\right)}_{t}}{{\left(\frac{A_{C}}{A_{R}}\right)}_{0}}$$

FTIR analysis was also intended to monitor the complete consumption of epoxy groups, a parameter directly linked to reduced migration risk in food-contact materials. Achieving minimal residual oxirane signals is critical for compliance with both EU and FDA regulations.

### Differential Scanning Calorimetry (DSC)

DSC scans were computed using DSC SDT 650 from TA Instruments (USA) using a standard closed aluminum pan under a synthetic air gas flow of 50 mL/min. The samples were heated from 25 to 300 °C, using the heating rates of 5, 10, 15, and 20 °C/min.

### Gel Content (%)

The gel content is a parameter that indicates the degree of conversion (cross-linking) achieved during the curing, it is calculated using eq 2. The cured samples (*M*_i_) were placed on a filter paper and immersed in a mixture of acetone and toluene using a Soxhlet apparatus for 48 h. Then, the residue was dried for 6 h until a constant weight (*M*_f_).

2 $$\mathrm{gel}\;\mathrm{content}\;\left(\%m\right)=\frac{M_{i}}{M_{i}-M_{f}}\times 100$$

### Swelling Ratio (%)

The swelling ratio (*S*_r_) was determined according to eq 3 and the results are shown as absorbed solvent content expressed in percentage units. Specimens with initial weight (*W*_i_), approximately 1 ± 0.01 g, were immersed in 50 mL of toluene for 24 h. Then, the specimens were gently dried to remove solvent excess and the final weights (*W*_f_) were recorded.

3 $$\mathrm{swelling}\;\mathrm{ratio}\;\left(S_{r}\;\mathrm{in}\%\right)=\frac{W_{f}-W_{i}}{W_{i}}\times 100$$

The swelling ratio in toluene was used to determine the cross-link density (*v*_d_) and the molar mass between cross-linking points (*M*c) of produced resins. According to Flory–Rehner theory,^34^ the cross-link density can be calculated using eq 4.

4 $$v_{d}=\frac{\ln\left(1-v_{p}\right)+v_{p}+X_{1}v_{p}^{2}}{v_{s}\left(\frac{v_{p}}{2}-v_{p}^{1/3}\right)}=\frac{1}{M_{c}}$$

where *v*_s_ is the solvent molar volume and *X*_1_ is the Flory–Huggins polymer–solvent interaction parameter, which for toluene is equal to 106.27 cm^3^·mol^–1^ and ∼0.391, respectively. The molar volume of the polymer *v*_p_ and can be calculated using eq 5.

5 $$v_{p}=\frac{\frac{1}{\rho_{\mathrm{polymer}}}}{\frac{S_{f}}{\rho_{\mathrm{solvent}}}+\frac{1}{\rho_{\mathrm{polymer}}}}$$

where ρ_polymer_ and ρ_solvent_ are the densities of the polymer (calculated from the relation between mass and volume of specimens) and that of the solvent, while *S*_f_ is the swelling factor. The *S*_f_ used in eq 5 was the unitary value of the swelling ratio (*S*_r_) calculated in eq 3.

In the context of food packaging, high gel content and low swelling are associated with superior barrier performance against moisture and gases, directly influencing food shelf life extension. Thus, these measurements are essential for assessing suitability for food-contact coatings and films.

### Curing Kinetics Modeling

The isoconversional models applied in this work are based on DSC analysis. In general, the conversion rate of a chemical reaction is expressed as the product of a temperature-dependent constant *k*(*T*) and a conversion function *f*(α) characteristics of the reaction mechanism, as

6 $$\frac{d\alpha }{dt}=k\left(T\right)\cdot f\left(\alpha \right)$$

The conversion rate assuming the constant rate *k*(*T*) defined by Arrhenius is shown in eq 7

7 $$k\left(T\right)=A\cdot \exp^{-E_{a}/\mathrm{RT}}$$

Considering the conversion rate in eq 6 for dynamic curing that dα/d*t* = β­(dα/d*T*), where β is the heating rate (K/min) and *k*(*T*) is the rate constant expressed by the temperature-dependent Arrhenius equation, eq 6 can be written as

8 $$\beta \frac{d\alpha }{dt}=A\cdot \exp^{-E_{a}/\mathrm{RT}}\cdot f\left(\alpha \right)$$

where *A* is the pre-exponential factor, *T* is the temperature, *E*_a_ is the activation energy dependent on α conversion and *R* = 8.314 J K^–1^ mol^–1^ is the universal gas constant.

Friedman’s model-free isoconversional method^35^ is originally obtained by the logarithmic form of the conversion rate shown in eq 8, as

9 $$\ln\left(\frac{d\alpha }{dt}\right)=\ln\left[A\cdot f\left(\alpha \right)\right]-\frac{E_{a}\left(\alpha \right)}{\mathrm{RT}}$$

The Vyazovkin method was developed to eliminate systematic errors in *E*_a_ when it varies with α, these errors occur mainly due to numerical instabilities and noisy interference caused using instantaneous rate values in the differential time dα/dt.^36,37^ Through an advanced nonlinear procedure that performs integrations over small conversion intervals, Δα ≈ 0.01, where *E*_a_ is considered constant, the Vyazovkin method is given

10 $$J\left[E_{a},T\left(t_{\alpha }\right)\right]=\int_{t\alpha -\Delta \alpha }^{t\alpha }\exp\left(\frac{{-E}_{a}}{\mathrm{RT}\left(t\right)}\right)dt$$

11 $$\sum_{i=1}^{n}\sum_{j\neq i}^{n}\frac{J\left[E_{a},T_{i}\left(t_{\alpha }\right)\right]}{J\left[E_{a},T_{j}\left(t_{\alpha }\right)\right]}$$

The Ozawa-Flynn-Wall (OFW) integral method uses the linear Doyle approximation^38^ of the function $p=\left(\frac{E_{a}}{\mathrm{RT}}\right)$ , where, by integration provides

12 $$\ln p\left(\frac{E_{a}}{\mathrm{RT}}\right)≅-5.331-1.052\frac{E_{a}}{\mathrm{RT}}$$

13 $$\ln\beta =\ln\frac{AE_{a}}{R\cdot g\left(\alpha \right)}-5.331-1.052\frac{E_{a}}{\mathrm{RT}}$$

The Kissinger-Akahira-Sunose (KAS) model-free method^39^ is obtained through the derivation of eq 8, this method corrects some deviations from simpler methods such as Ozawa-Flynn-Wall by applying the Coats-Redfern approximation,^41^ written as

14 $$\ln\left(\frac{\beta }{T^{2}}\right)=\ln\left(\frac{A\cdot R}{E_{a}\cdot g\left(\alpha \right)}\right)-\frac{E_{a}}{\mathrm{RT}}$$

## Results and Discussion

### Fourier Transform Infrared Spectroscopy (FTIR) measurements

FTIR analyses were performed for ESO/CA, ESO/MA, and ESO/SA systems to evaluate the curing process as a function of time. During curing, aliquots were periodically removed and analyzed by FTIR. The results are presented in Figures 2, 3, and 4.

**Figure 2:**
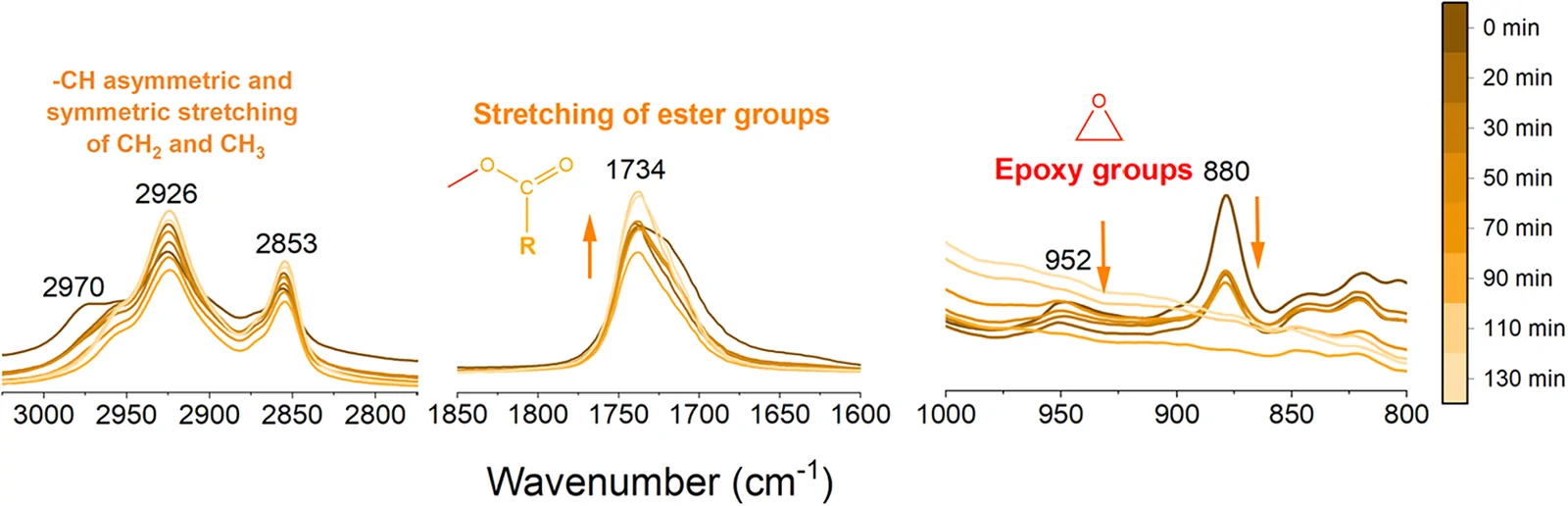
FTIR spectra of the indicated compositions ESO/CA. At 110 °C. Time indicated.

**Figure 3:**
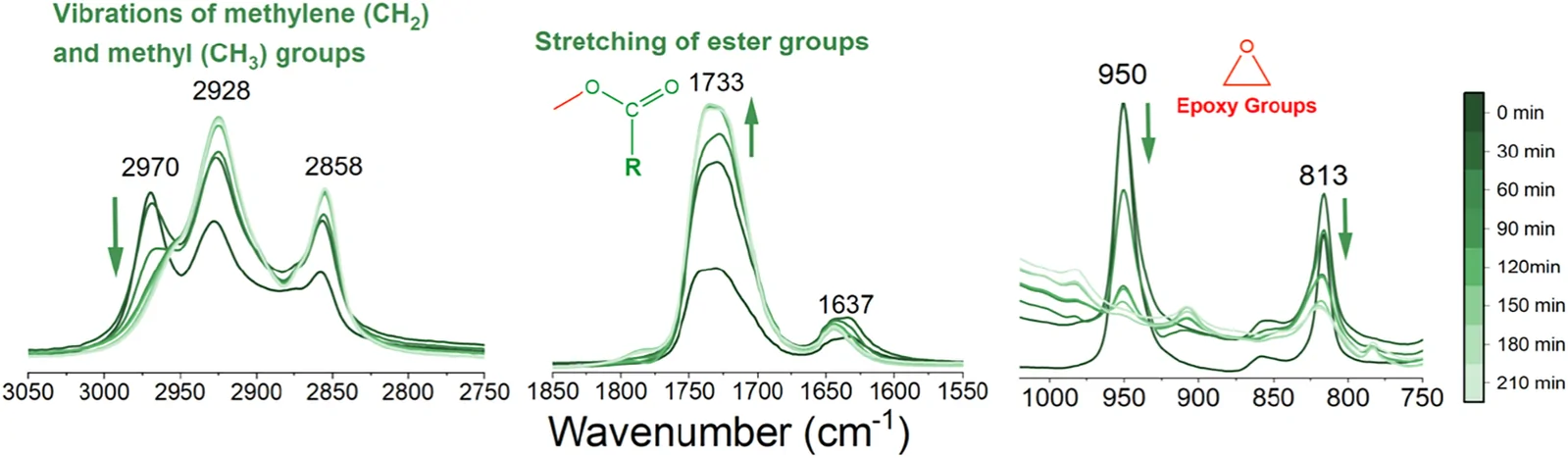
FTIR spectra of the indicated compositions ESO/MA. At 110 °C. Time indicated.

**Figure 4:**
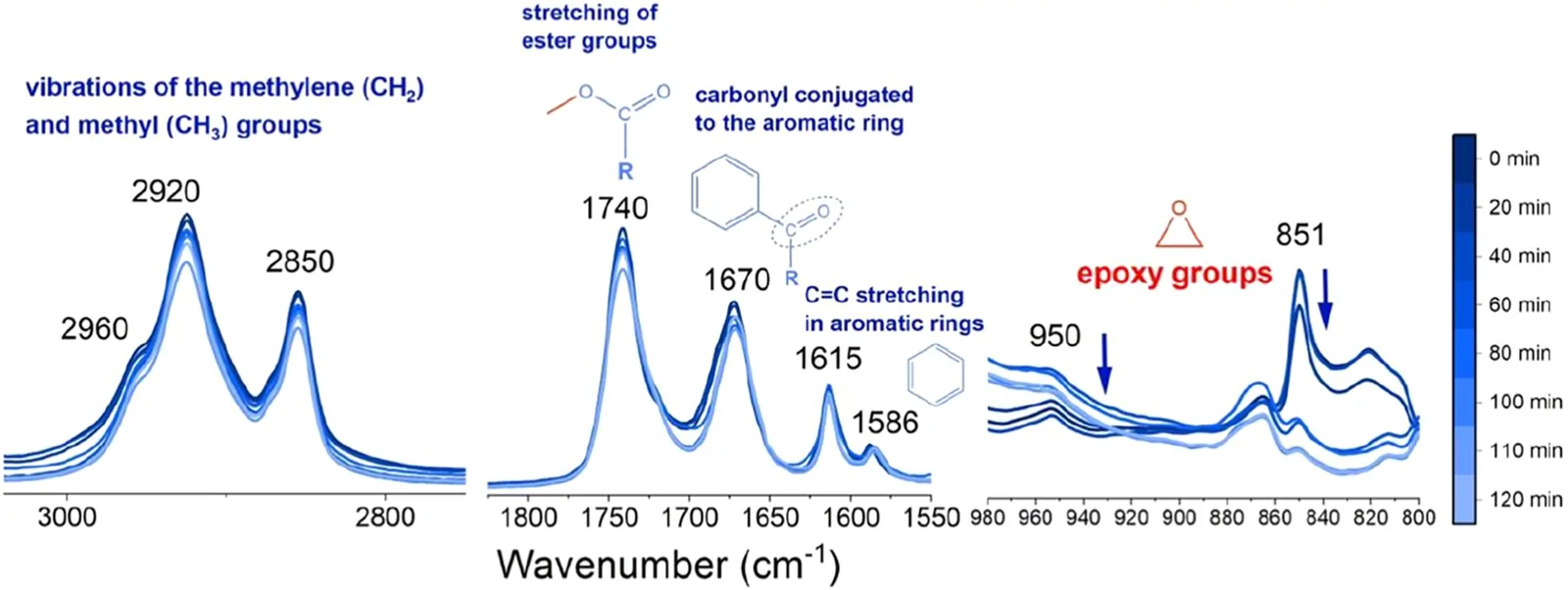
FTIR spectra of the indicated compositions ESO/SA. At 110 °C. Time indicated.

Figure S1 (Supporting Information) shows the FTIR spectra of the ESO/CA system at different curing times. The spectra presented in Figure 2 display characteristic absorption bands associated with hydroxyl, aliphatic, carbonyl, and epoxy groups. The broad band centered at approximately 3420 cm^–1^ (Figure S1) corresponds to O–H stretching vibrations associated with hydroxyl groups.^40^ Changes in the intensity of this band throughout the curing process reflect the evolution of the reaction and the development of the cross-linked polymer network. Citric acid (CA), which contains three carboxylic acid groups, can react with multiple epoxy groups from ESO, promoting the formation of a highly cross-linked three-dimensional structure.

The peaks located at 2970 cm^–1^, 2926 cm^–1^, and 2853 cm^–1^ correspond to the asymmetric and symmetric stretching vibrations of methylene (CH_2_) and methyl (CH_3_) groups.^41,42^ These bands remain relatively constant during curing, indicating that the aliphatic backbone of the ESO chains is largely preserved during network formation. The absorption band at 1734 cm^–1^, assigned to C=O stretching of ester groups, increases in intensity over time, confirming the formation of ester linkages during the reaction between the epoxy groups of ESO and the carboxylic groups of CA.^32,40,43^ Simultaneously, the gradual decrease and eventual disappearance of the epoxy-related bands at 952 cm^–1^ and 880 cm^–1^ indicate epoxy ring opening and progression of the curing reaction.^7,8,21,25,40,44^

Figure S2 (Supporting Information) presents the FTIR spectra of the ESO/MA system at different curing times. Figure 3 shows enlarged views of the main absorption bands. The peaks at 2970 cm^–1^, 2928 cm^–1^, and 2858 cm^–1^ correspond to the asymmetric and symmetric stretching vibrations of methylene (CH_2_) and methyl (CH_3_) groups. During curing, the band at 2970 cm^–1^ gradually decreases in intensity, which may be associated with changes in the local chemical environment caused by the formation of new bonds within the developing polymer network.^40^ Chemical modifications occurring during curing, such as ester bond formation, may influence the vibrational behavior of nearby C–H bonds by altering their electronic environment.^7^

The absorption band at 1733 cm^–1^ corresponds to the C=O stretching vibration of ester groups, while the bands at 950 cm^–1^ and 813 cm^–1^ are characteristic of epoxy groups.^42,46^ Although the increasing intensity of the carbonyl band at 1733 cm^–1^ confirms the occurrence of esterification reactions, the persistence of the epoxy bands indicates incomplete epoxy conversion under the curing conditions employed.^45^

The incomplete curing behavior observed for ESO/MA can be attributed to the molecular structure and reactivity of maleic acid. The two carboxylic groups in maleic acid are constrained by a rigid C=C double bond, evidenced by the absorption at 1637 cm^–1^, which limits their spatial accessibility and effective orientation toward epoxy groups. As a result, the formation of monoester structures may be favored, leaving a fraction of oxirane groups unreacted.^44,45^

In addition, the acid–epoxy reaction is known to proceed relatively slowly in the absence of catalysts and at moderate curing temperatures. Diffusion limitations arising after the onset of gelation may further hinder complete epoxy conversion. The need to predissolve maleic acid in isopropyl alcohol to achieve homogenization with ESO may also influence the early stages of curing, delaying effective acid–epoxy interactions. Furthermore, partial decarboxylation of maleic acid during thermal treatment may reduce the availability of carboxylic groups for cross-linking. These combined factors contribute to the reduced cross-linking efficiency observed in the ESO/MA system.

Figure S3 (Supporting Information) presents the FTIR spectra of the ESO/SA system at different curing times, while Figure 4 shows the corresponding enlarged spectral regions. The spectra display a broad absorption band between 3600 cm^–1^ and 3180 cm^–1^ associated with hydroxyl groups (O–H). At the beginning of curing, this band exhibits two distinct contributions: one near 3500 cm^–1^, corresponding to the phenolic hydroxyl groups of salicylic acid, and another near 3250 cm^–1^ associated with the O–H stretching of carboxylic acid groups (COOH).^47–49^ The carboxylic O–H band initially shows higher intensity due to strong hydrogen-bonding interactions.^41,45,50,51^

The peaks at 2960 cm^–1^, 2920 cm^–1^, and 2850 cm^–1^ correspond to the asymmetric and symmetric stretching vibrations of methylene (CH_2_) and methyl (CH_3_) groups and remain relatively unchanged during curing, indicating that the aliphatic structure of the ESO backbone is preserved. The absorption band at 1740 cm^–1^ corresponds to the C=O stretching of ester groups formed during curing, while the band at 1670 cm^–1^ is attributed to the carbonyl group conjugated with the aromatic ring of salicylic acid. The band at 1740 cm^–1^ shows a modest reduction in intensity during curing, which may be related to changes in the local chemical environment caused by hydrogen bonding and network formation.^7,8,21,25,30,52^

The peaks at 1615 cm^–1^ and 1586 cm^–1^ correspond to the aromatic C=C stretching vibrations of salicylic acid. The persistence of these bands indicates that the aromatic structure remains intact during curing, contributing to the rigidity and stability of the resulting polymer network.^25,26,28^ Finally, the gradual decrease in the intensity of the epoxy band at 950 cm^–1^ confirms the opening of epoxy rings and the formation of the cross-linked network through reaction with the carboxylic groups of salicylic acid.

Although FTIR analysis was primarily used qualitatively to monitor curing progression, the persistence of epoxy-related bands at 950 cm^–1^ and 813 cm^–1^ after curing provides clear evidence of residual oxirane groups in the ESO/MA system. Compared with the ESO/CA and ESO/SA systems, the relatively higher intensity of these bands indicates incomplete epoxy conversion and reduced cross-linking efficiency under the catalyst-free curing conditions employed.

Using the reference band (*A*_*R*_)_0→*t*_ and one associated with epoxy reactive functional groups (*A*_*C*_)_0→*t*_, it is possible to evaluate the relative degree of cure conversion using the Beer–Lambert Law, the results are presented in Figure 5.

**Figure 5:**
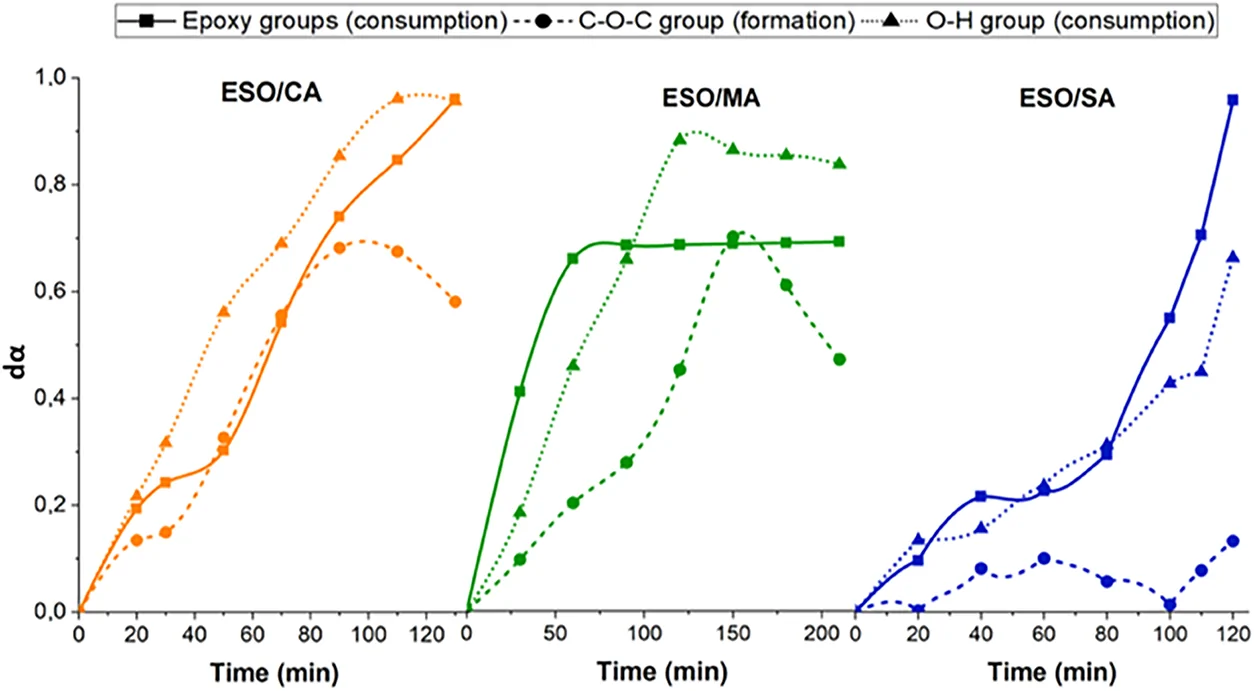
Relative degree of conversion (dα) as a function of time (minutes) is estimated by the Beer–Lambert Law.

ESO/CA achieves a complete conversion of epoxy groups (dα = 0.965) in approximately 100 min, indicating that CA is a highly effective curing agent for ESO. The rapid conversion rate suggests that CA promotes efficient curing, probably due to its expected good reactivity with epoxy groups and adequate thermal stability. Furthermore, the consumption of O–H groups observed in the curves is a strong indication that the curing reaction is progressing.

ESO/MA has a much slower conversion rate and does not reach full conversion even after 200 min. This data confirms that MA is not effective as a curing agent for ESO under the tested conditions. The low conversion indicates that MA, possibly due to its thermal instability, the proximity of carboxylic acids, and the possibility of undergoing decarboxylation, does not react efficiently with epoxy groups. MA appears to be the least effective of the three acids tested to promote ESO curing. Its low conversion can be attributed to the combination of low reactivity and possible thermal degradation before curing can be completed. Comparison with other acids highlights that MA cannot maintain the integrity necessary for efficient curing, resulting in incomplete cross-linking and, therefore, a material with inferior thermal properties. The behavior of the curve, where the consumption of the epoxy groups stabilizes after 50 min while the O–H groups continue to be consumed, indicates a difference in the reactivity and accessibility of the groups involved in the curing. Initially, the more reactive epoxy groups react quickly with the O–H groups, and then the reaction may be limited by lower reactivity or diffusion difficulties of the remaining epoxy groups. Furthermore, the O–H groups may be involved in other secondary reactions, continuing to be consumed even after the stabilization of the epoxy groups.

ESO/SA has a high conversion, reaching dα close to 1, although slightly slower than ESO/CA. This suggests that SA is an effective curing agent, almost as efficient as CA, but with somewhat slower kinetics. The thermal stability of SA and its reactivity with epoxy groups are sufficient to promote a nearly complete cure.

The profile of the consumption curves of the epoxy groups for ESO/CA and ESO/SA represents a multimodal reaction, with the presence of two distinct stages in the curve, suggesting that the curing occurs in two phases. In the first, some of the epoxy groups react quickly, possibly due to their high reactivity with the O–H groups available in greater quantities at the beginning of the reaction. In the second phase, the remaining epoxy groups react slower which may be due to a decrease in the concentration of the most reactive reactants or the need for a structural rearrangement within the material that facilitates the reaction of the remaining epoxy groups. This is consistent with dα ≈ 0.965 for ESO/CA and dα close to 1.0 for ESO/SA from the Beer–Lambert analysis (epoxide band at ∼950 cm^–1^).

In the case of ESO/SA, despite effective epoxy consumption, the FTIR spectra indicates a lower formation of C–O–C bonds compared to ESO/CA. This suggests that the curing mechanism in ESO/SA is strongly influenced by the presence of salicylic acid, which contains a highly reactive phenolic −OH group. The high nucleophilicity of this group enables rapid epoxide ring opening, favoring direct epoxy–OH interactions over esterification with the carboxyl (−COOH) groups. As a result, the formation of ester (C–O–C) bonds is less prominent in ESO/SA than in ESO/CA, where acid-mediated esterification is more dominant. Additionally, the lower intensity of the carbonyl stretching band (1740 cm^–1^) reinforces the hypothesis that ester bond formation is not as predominant in this system. Instead, the curing process in ESO/SA likely leads to a polymeric network enriched with hydroxyl groups, which could influence the material’s thermal stability, hydrophilicity, and mechanical performance. The presence of these hydroxyl-rich structures may also contribute to alternative cross-linking pathways, such as hydrogen bonding interactions, further distinguishing ESO/SA from other systems. This hydroxyl-richer network helps explain the higher elongation and lower modulus measured in ESO/SA.

### Gel Content and Swelling Ratio

The gel content in toluene and acetone (Figure 6a) was used as a parameter to verify the amount of monomers participating in the cross-linking reaction. The findings indicated that ESO/CA and ESO/SA exhibited similar values, exceeding 80%, which confirms the presence of highly cross-linked networks. In the context of food chemistry, gel content above 80% and corresponding high cross-link density imply lower diffusivity of small molecules, including moisture and oxygen, which is a key property for barrier materials in food packaging. Swelling resistance in toluene also indirectly suggests reduced swelling in edible oils or fatty foods, thus enhancing suitability for food-contact coatings.

**Figure 6:**
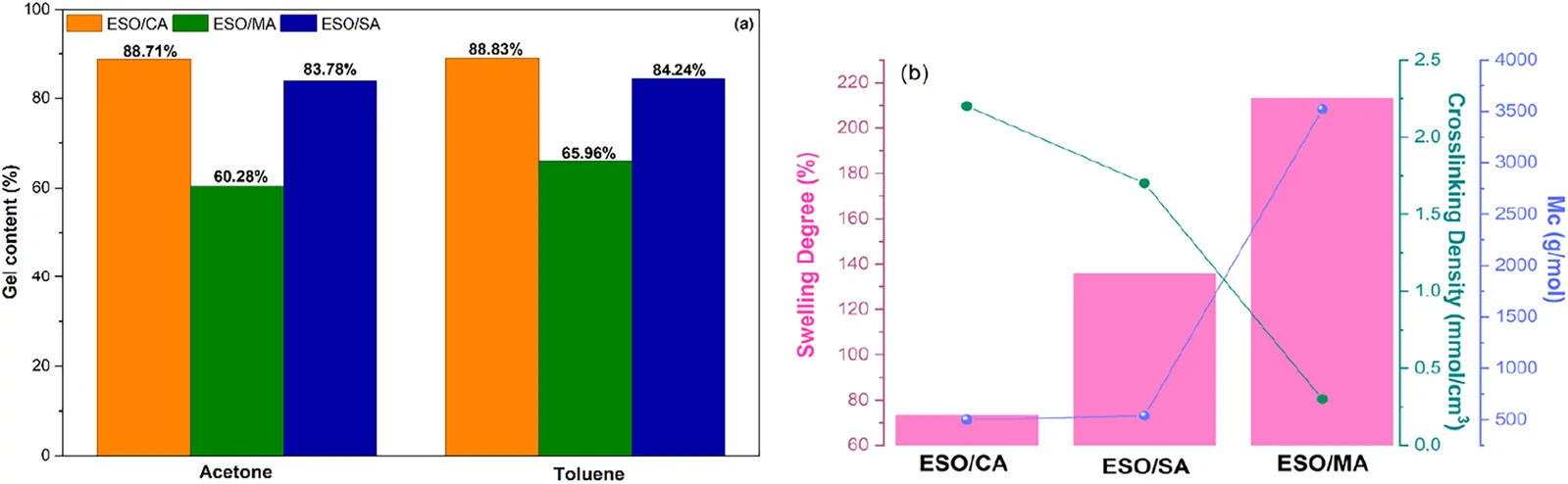
(a) The gel content of epoxy resins in acetone and toluene, and (b) the influence of the carboxylic acid type on the swelling rate in toluene, cross-link density (υ_*d*_) and molar mass between cross-linking points (Mc).

Conversely, the lower gel content observed in ESO/MA implies that the network may contain non-cross-linked chains, such as unreacted epoxy group residues remaining after curing, which could increase the soluble fraction and thus reduce the gel content. This aligns with the FTIR results from this study, where ESO/MA showed low epoxy group absorption even after curing.

The swelling degree in toluene, along with its correlation to cross-link density (*v*_d_) and molar mass between chain segments (*M*_c_), was measured (Figure 6b). ESO/CA and ESO/SA exhibited cross-link density values like those reported in the literature,^55,56^ ranging from 1.7 to 2.1 mmol/cm^3^. ESO/CA showed the highest cross-link density, consistent with gel content data, due to its structure containing three carboxylic groups, leading to a densely cross-linked network that prevents the solvent penetration and dissolution of the resin. In comparison, CA’s tighter polymer chains result in a more rigid structure with less expansion capacity than MA and SA. ESO/SA contains a benzene ring, which reduces chain mobility, its cross-link density was lower than ESO/CA, around 1.7, it has one carboxylic group. This leaves spaces in the structure after curing, allowing solvent penetration. ESO/MA exhibited the lowest cross-link density at approximately 0.3, indicating significant solvent penetration due to the low degree of cross-linking.

### Differential Scanning Calorimetry (DSC) Measurements

Figure S4 (Supporting Information) presents the DSC thermograms obtained for ESO/CA (a), ESO/MA (b), and ESO/SA (c) at different heating rates (5, 10, 15, and 20 °C min^–1^). The maximum curing rate (*C*_max_), curing enthalpy (Δ*H*), and characteristic temperatures (*T*_0.01_, *T*_p_, and *T*_0.99_) were calculated from the exothermic peaks observed for the ESO/CA and ESO/SA systems and are summarized in Table 1. For these systems, the reported values correspond to the first and second exothermic peaks identified in the DSC thermograms.

**Table 1:** Curing Parameters for ESI/CA and ESO/SA^a^

			ϕ °C/min			
peak	sample code	parameter	**5**	**10**	**15**	**20**
1	ESO/CA	*C*_max_ (min^–1^)	0.128	0.207	0.487	0.792
		*T*_0.01_ (°C)	58.42	65.63	75.32	83.45
		*T*_p_ (°C)	111.71	124.87	132.76	143.98
		*T*_0.99_ (°C)	177.35	183.48	194.83	202.98
	ESO/SA	*C*_max_ (min^–1^)	0.042	0.094	0.179	0.387
		*T*_0.01_ (°C)	66.72	76.46	83.23	89.62
		*T*_p_ (°C)	141.86	157.41	164.20	169.55
		*T*_0.99_ (°C)	179.91	197.35	204.45	213.05
2	ESO/CA	*C*_max_ (min^–1^)	0.025	0.165	0.177	0.204
		*T*_0.01_ (°C)	177.35	183.48	194.83	202.98
		*T*_p_ (°C)	185.73	203.76	212.44	216.98
		*T*_0.99_ (°C)	194.17	213.45	224.63	228.20
	ESO/SA	*C*_max_ (min^–1^)	0.183	0.222	0.437	0.498
		*T*_0.01_ (°C)	185.35	200.57	207.62	215.02
		*T*_p_ (°C)	229.96	245.60	252.65	260.20
		*T*_0.99_ (°C)	242.41	258.55	268.55	275.73

a C_max_ maximum cure rate. *T*_0.01_ temperature at 0.01° of relative conversion (assumed as the start of curing). *T*_p_ peak temperature. *T*_0.99_ temperature at 0.999° of relative conversion (assumed as the end of curing).

The DSC curves of the ESO/CA and ESO/SA systems exhibit two distinct exothermic peaks. The first peak corresponds to the primary curing reaction, where most of the epoxy groups from ESO react with the carboxylic groups of the curing agents, leading to the formation of ester linkages and the development of the cross-linked polymer network. This stage is typically associated with the main epoxy–acid reaction and is highly exothermic due to the formation of the three-dimensional network structure.^19^

The second exothermic peak appears at higher temperatures and is generally attributed to secondary curing processes occurring after the primary cross-linking stage. These reactions may involve additional epoxy–acid interactions, rearrangements within the partially formed network, or transformations of intermediate species generated during the initial curing step.^7,8,26,28,46,52,53^ Such secondary reactions commonly produce smaller and less intense exothermic peaks in DSC thermograms.^54^

Analysis of the data in Table 1 indicates that increasing the heating rate leads to higher curing rates and shifts the peak temperatures (*T*_p_) toward higher values. For the ESO/CA system, *T*_p_ increased by approximately 32 °C when the heating rate was raised from 5 to 20 °C min^–1^, while a shift of approximately 29 °C was observed for the ESO/SA system. Differences in curing behavior can be related to the molecular structure of the curing agents. Citric acid contains three carboxylic groups capable of reacting with epoxy groups, which facilitates network formation and promotes higher reactivity. In contrast, salicylic acid contains only one carboxylic group and an aromatic ring, which may reduce molecular mobility and limit the accessibility of reactive sites, resulting in curing reactions occurring at higher temperatures.

The DSC thermograms of the ESO/MA system (Figure S4b) differ significantly from those observed for the other systems. No well-defined exothermic peaks were detected within the investigated heating-rate range. Instead, a gradual increase in the heat-flow baseline at higher temperatures was observed, indicating that the curing reaction proceeds only to a limited extent under the applied conditions. This behavior contrasts with typical epoxy–acid curing systems, in which pronounced exothermic peaks are associated with epoxy ring opening and cross-linked network formation.

These observations are consistent with the FTIR results, which showed persistent oxirane absorption bands after thermal treatment, indicating incomplete epoxy conversion in the ESO/MA system. The limited curing efficiency can be attributed to the molecular structure of maleic acid. The two carboxylic groups of maleic acid are constrained by a rigid C=C double bond, as evidenced by the absorption band at 1637 cm^–1^, which restricts their spatial accessibility and orientation toward epoxy groups. As a result, the formation of monoester structures may be favored, leaving a fraction of oxirane groups unreacted.^44,45^

Thermogravimetric analysis (TGA) of maleic acid (Figure S5, Supporting Information) further supports this interpretation. Maleic acid begins to undergo mass loss at temperatures around 150 °C, associated with dehydration and decarboxylation reactions that reduce the availability of carboxylic groups required for effective cross-linking. Although the curing temperature used in this study (110 °C) is below the main decomposition temperature, the onset of thermal degradation or side reactions may influence the curing process. Overall, the combined FTIR, DSC, and TGA results indicate that maleic acid exhibits limited reactivity as a curing agent for ESO under the catalyst-free curing conditions employed.

### Curing Steps of ESO/CA and ESO/SA

ESO curing using CA and SA as curing agents occurs mainly in two stages: initiation and propagation,^9,10^ as illustrated in Figure 7. In the initiation stage, the reaction proceeds through an acid-catalyzed epoxide ring-opening mechanism. Epoxide oxygen is first protonated by the carboxylic acid, increasing the electrophilicity of the oxirane ring. This protonation facilitates the opening of the epoxy ring, generating a carbocation-like intermediate. Subsequently, the carboxylate species attacks this electrophilic center, leading to the formation of a β-hydroxy ester intermediate and stabilizing the structure through proton exchange.^32,56^

**Figure 7:**
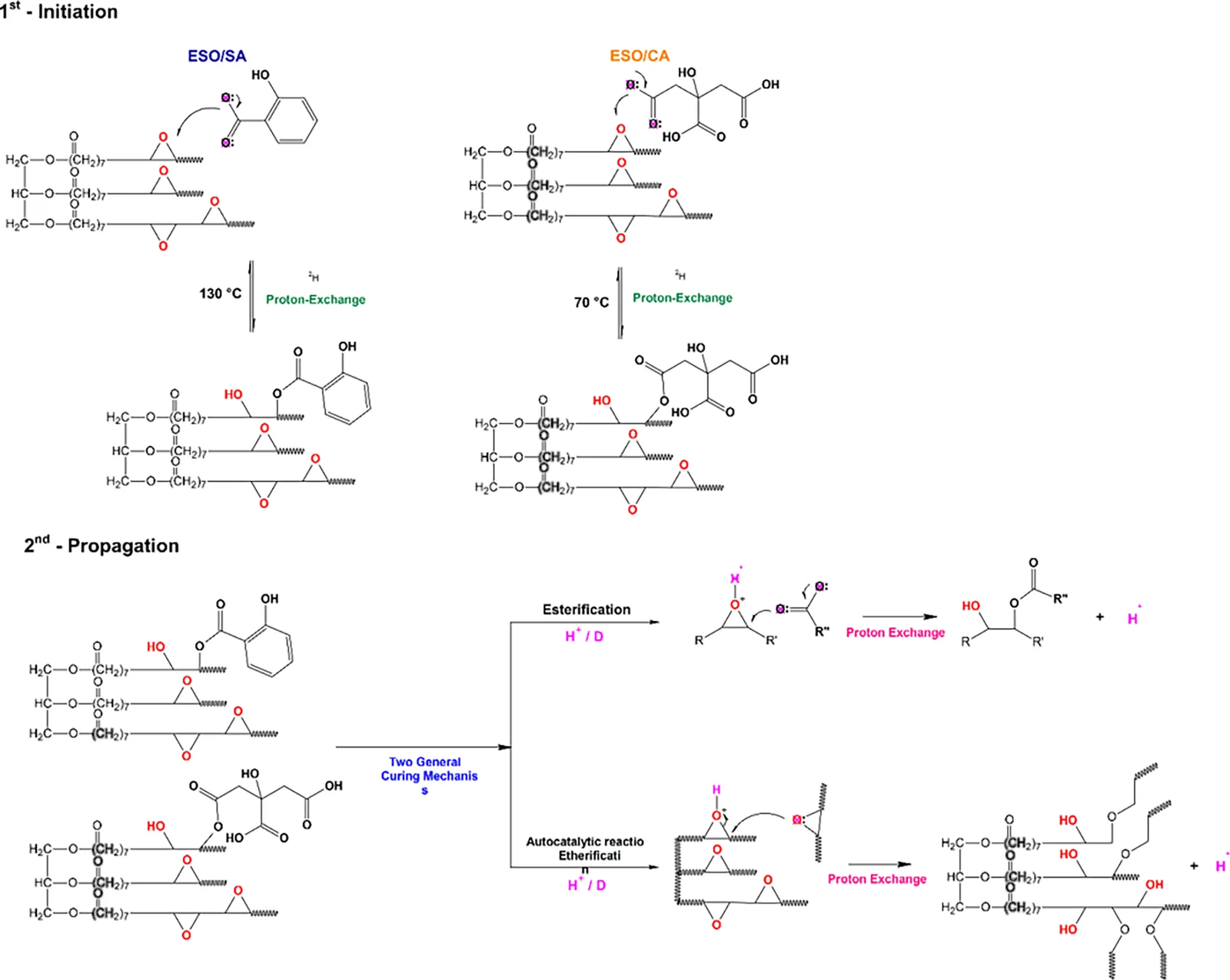
Curing of epoxidized soybean oil with CA and SA as curing agents.

In the propagation stage, the reaction proceeds mainly through esterification reactions, in which hydroxyl groups (−OH) formed during the initiation step attack other available epoxy groups. This promotes further epoxide ring-opening reactions and the formation of additional ester linkages. During this process, proton exchange occurs, regenerating the acidic species that continues to catalyze the reaction. As the reaction progresses, the polymer network begins to form as epoxy groups react with carboxyl groups, linking the ESO chains through ester bonds.

In addition, autocatalytic reactions and etherification may occur during propagation, contributing to the development of the three-dimensional polymer network. In these reactions, hydroxyl groups generated during curing react with additional epoxy groups, forming ether (−O−) linkages that further increase the cross-link density of the polymer matrix.^19,53,57^

The resulting network structure depends on the carboxylic acid used. In the case of SA, the resulting network tends to be less densely cross-linked, leading to a matrix with greater flexibility and specific properties associated with the presence of the aromatic ring.^58^ In contrast, CA, which contains three carboxyl groups, promotes a higher cross-linking density, resulting in a more rigid and thermally stable three-dimensional polymer network.^50,52^

Therefore, the curing of these epoxy systems leads to polymeric networks with tunable properties depending on the structure and functionality of the carboxylic acids employed, enabling the development of materials with tailored characteristics for different applications.

### Curing Kinetics Modeling of ESO/CA and ESO/SA

The curing kinetics of polymers is a key research area for understanding the progression of curing reactions under different thermal conditions and for optimizing material properties and processing parameters. Curing kinetics provides important information about reaction rates and apparent activation energies, offering mechanistic insight into the different curing stages previously described for ESO/CA and ESO/SA systems. In this study, curing kinetics of epoxidized soybean oil (ESO)–based epoxy systems were investigated using differential scanning calorimetry (DSC) combined with model-free isoconversional methods. Model-free isoconversional methods were deliberately employed in this study, particularly the Friedman and Vyazovkin approaches, as they do not require the assumption of a predefined reaction model *f*(α) or the determination of a single pre-exponential factor A. This strategy is especially suitable for epoxy–acid curing systems, which typically involve multiple overlapping reaction mechanisms and diffusion-controlled stages as curing progresses. By avoiding oversimplified kinetic assumptions, the isoconversional analysis allows a more realistic description of the evolution of *E*_a_ as a α. The kinetic analysis was performed for ESO/CA and ESO/SA systems, while ESO/MA was excluded due to the absence of effective curing, as evidenced by FTIR and DSC results.

DSC heat flow data collected at multiple heating rates (5–20 °C/min) were converted into the degree of conversion by normalizing the partial reaction enthalpy at a given temperature to the total curing enthalpy. This approach allows the comparison of reaction kinetics at identical conversion levels under different thermal conditions, forming the basis of isoconversional kinetic methods such as Friedman and Vyazovkin. The apparent *E*_a_ as a α was determined and is presented in Figure 8.

**Figure 8:**
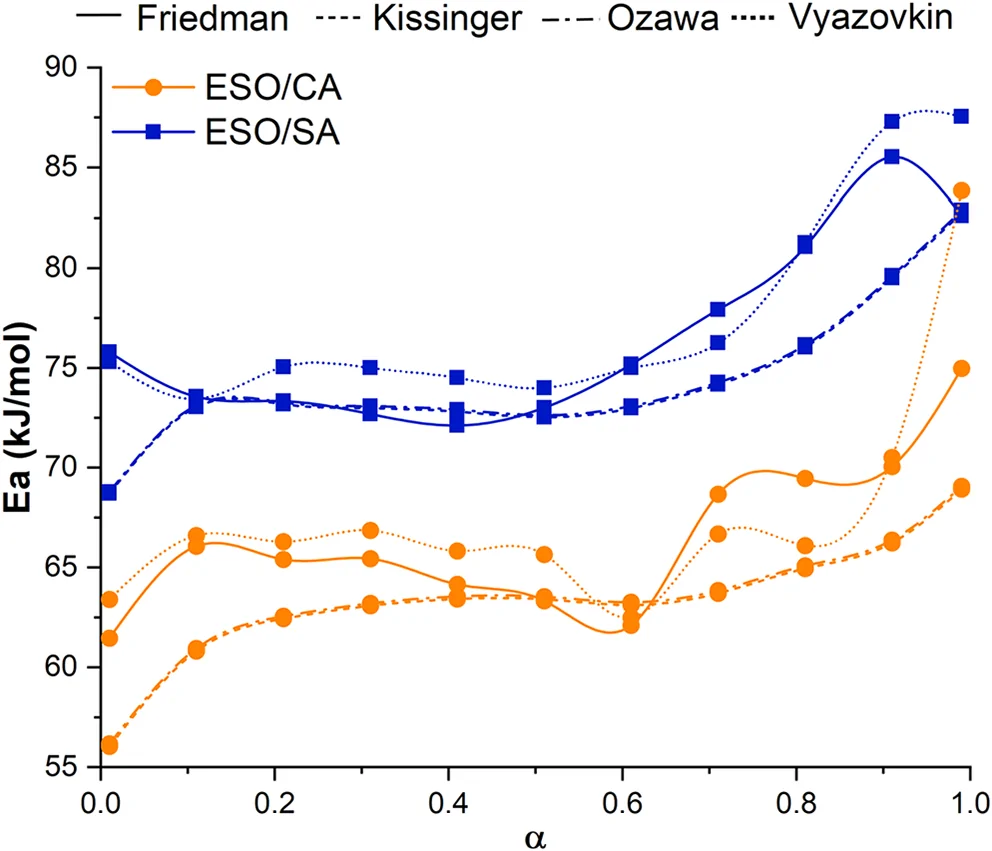
Kinetic parameters of the Friedman Isoconversional, Kissinger, Ozawa, and Vyazovkin model *E*_a_ versus α.

Among the kinetic approaches applied, the Friedman and Vyazovkin methods exhibited excellent correlation coefficients (*R* ^2^ > 0.99), confirming their suitability for describing the curing behavior of ESO/CA and ESO/SA systems. In contrast, the Ozawa-Flynn-Wall and Kissinger-Akahira-Sunose methods showed lower correlation coefficients (*R* ^2^ ranging from 0.81 to 0.88), indicating limited applicability for detailed kinetic interpretation of these complex curing reactions.

For the ESO/CA formulation, both Friedman and Vyazovkin methods revealed similar *E*_a_ profiles. An initial increase in *E*_a_ was observed at low conversion levels (α < 0.11), from 61 to 67 kJ/mol (Friedman) and from 64 to 67.3 kJ/mol (Vyazovkin), followed by a more pronounced increase at intermediate conversion (0.11 < α < 0.61) and a continued rise at higher conversion levels (α > 0.61), reaching a maximum of approximately 85 kJ/mol. This behavior reflects the transition from chemically controlled reactions to diffusion-limited stages associated with network formation.

The ESO/SA system exhibited consistently higher Ea values throughout the conversion range. An initial decrease in *E*_a_ (from 76 to 73 kJ/mol) was observed, followed by a plateau at intermediate α (0.11 < α < 0.51) and a final increase at higher conversion levels (α > 0.51), reaching approximately 87 kJ/mol. The higher *E*_a_ observed for ESO/SA are attributed to the aromatic structure of salicylic acid and its capacity for hydrogen bonding, which increase network rigidity and impose additional energetic barriers to curing progression.^59^

Figure 8 illustrates the variation of the apparent *E*_a_ as a α for the ESO/CA and ESO/SA systems, as determined using different kinetic approaches. The variation of *E*_a_ with conversion clearly demonstrates that the curing reactions of ESO/CA and ESO/SA are multistep processes involving changes in reaction mechanisms and increasing diffusion limitations at advanced stages of curing. In this context, single-*E*_a_ models such as Kissinger and Ozawa, which assume constant activation energy throughout the reaction, cannot capture the complexity of these systems. Although these models are often employed for preliminary kinetic assessments,^39,60^ they provide only global apparent *E*_a_ values and fail to describe the evolution of curing kinetics as a function of conversion.^61–64^ Therefore, in this work, the Friedman and Vyazovkin methods are considered the most reliable tools for interpreting the curing behavior of ESO-based epoxy systems. The observed variations in *E*_a_ with conversion are consistent with the curing stages described in Figure 7, reflecting the transition from chemically controlled epoxy–acid reactions to diffusion-limited processes at higher conversion levels.

Figures 9 and 10 illustrate the theoretical and experimental values for the relative degree of conversion as a function of the Temperature (Friedman and Vyazovkin respectively). At Figures S9 and S10 (Supporting Information) illustrate the theoretical and experimental values of the Ozawa, and Kissinger models for ESO/CA and ESO/SA, respectively. A satisfactory fit was obtained for all compounds, with average errors of less than 3% for Friedman, and discrepancies of less than 5% for Vyazovkin, and *R* ^2^ values (Table 2) close to 1, indicating that Friedman’s and Vyazovkin’s model describes the curing mechanism well for the samples studied. Ozawa and Kissinger models displayed discrepancies greater than 8% for ESO/CA and 13% for ESO/SA – Ozawa, and discrepancies greater than 10% for ESO/CA and 18% for ESO/SA – Kissinger, indicating that the Ozawa and Kissinger models do not describe the curing mechanism well for the samples studied.

**Table 2:** Statistical Linear Correlation (*R* ^2^) Parameters Computed Using Friedman, Ozawa, Kissinger, and Vyazovkin xModels

sample code	Friedman	Ozawa	Kissinger	Vyazovkin
ESO/CA	0.99965	0.88761	0.85765	0.99033
ESO/SA	0.99921	0.85674	0.81342	0.99007

**Figure 9:**
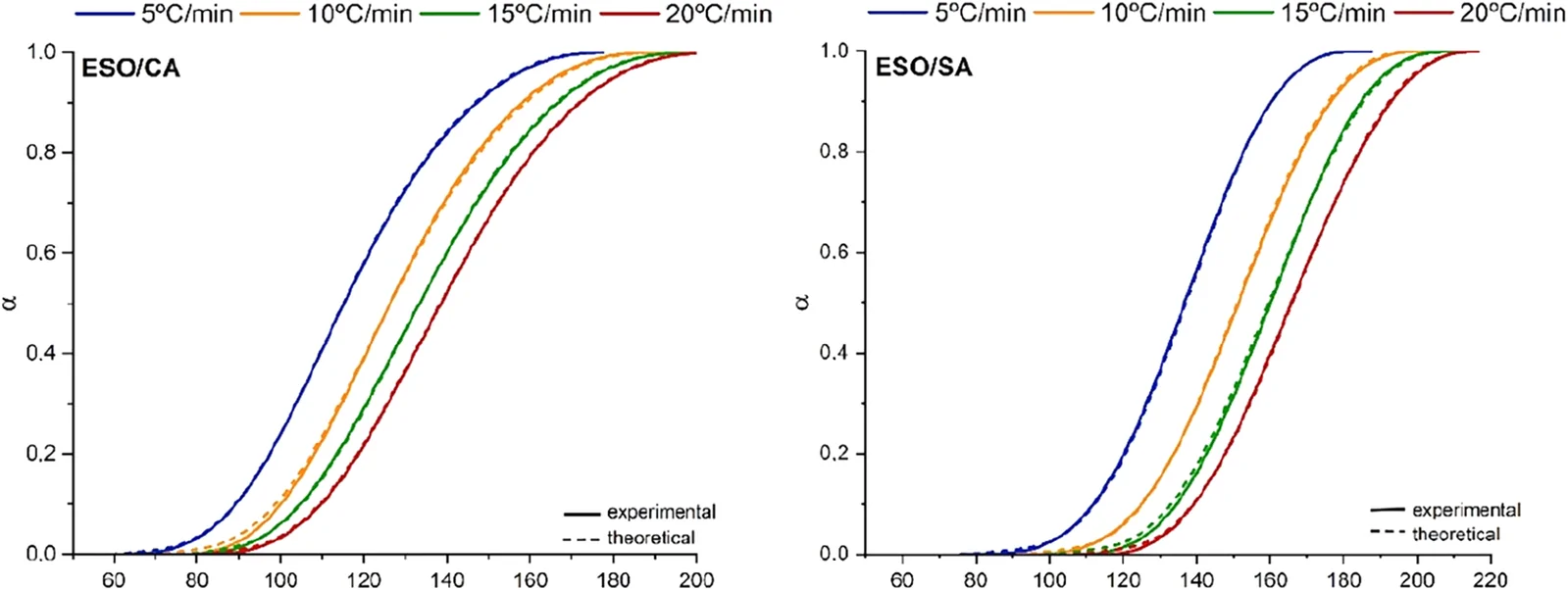
Comparison between experimental values (lines) and theoretical values (dotted) of the relative degree of conversion as a function of temperature estimated by the Friedman model for ESO/CA and ESO/SA at the indicated rates. *R* ^2^ > 0.99 and mean error <3% for Friedman.

**Figure 10:**
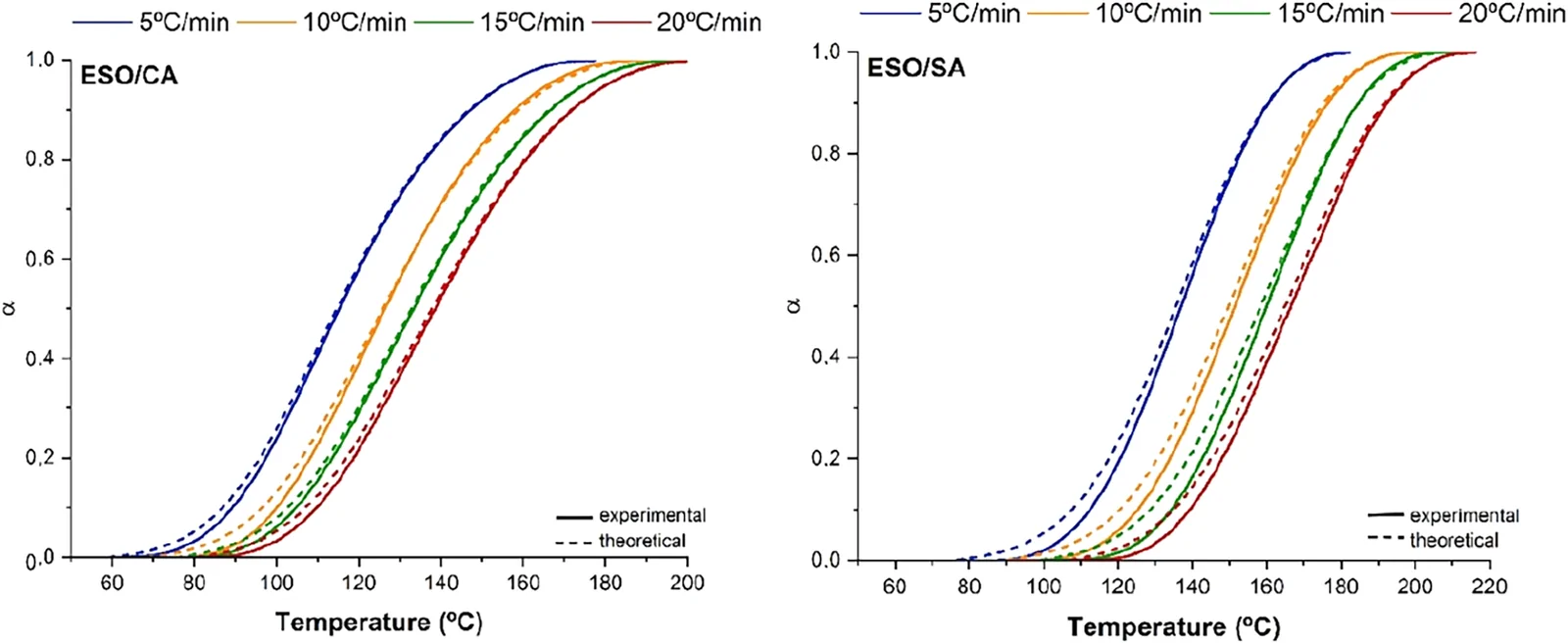
Comparison between experimental values (lines) and theoretical values (dotted) of the relative degree of conversion as a function of temperature estimated by the Vyazovkin model for ESO/CA and ESO/SA at the indicated rates. Vyazovkin: *R* ^2^ ≈ 0.99 and discrepancies <5%. Ozawa and KAS showed lower fits (*R* ^2^ < 0.88) in these systems.

### Tensile Mechanical Properties

The mechanical behavior of polymeric networks synthesized from ESO cured with salicylic acid (SA) and citric acid (CA) is presented in Figure 11. These systems exhibit distinct mechanical responses that can be directly correlated with the chemical structure and functionality of the curing agents used in thermoset formation.

**Figure 11:**
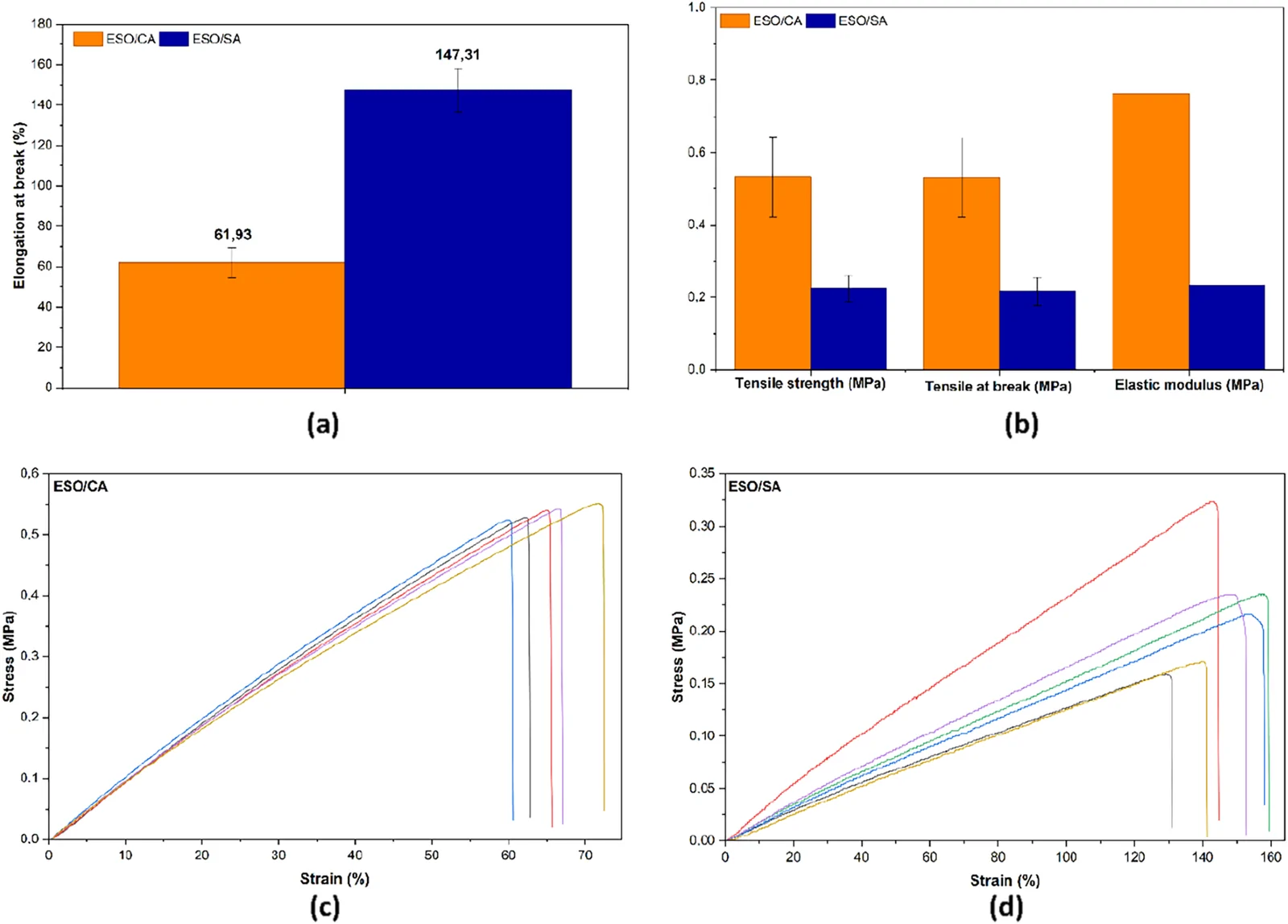
Tensile mechanical properties for ESO/CA and ESO/SA: (a) elongation at break; (b) tensile strength, tensile ate break and elastic modulus; (c) and (d) stress versus strain curves.

SA is an aromatic monocarboxylic acid containing one carboxylic group and one phenolic hydroxyl group. Under thermal curing conditions, the carboxylic acid group reacts with the oxirane rings of ESO through ring-opening esterification, forming covalent cross-links. However, due to its limited functionality, SA promotes the formation of a network with relatively low cross-linking density. Additionally, the presence of the rigid aromatic ring does not favor extensive branching or the formation of a highly interconnected three-dimensional network. As a result, the resulting polymer network exhibits higher chain mobility and a less compact structure.

In contrast, CA is a multifunctional aliphatic compound containing three carboxylic acid groups and one hydroxyl group. Its higher functionality enables multiple covalent interactions with epoxy groups from ESO, leading to the formation of a denser and more highly cross-linked polymeric network. This increased cross-linking restricts the segmental motion of polymer chains and results in a more rigid and mechanically robust material.

These structural differences are reflected in the mechanical performance of the materials. The tensile stress–strain curves show that ESO/SA exhibits significantly greater elongation at break, reaching average values of 147.31%, compared with 61.93% for ESO/CA. This behavior is consistent with the more flexible and loosely cross-linked network formed in the ESO/SA system, which allows extensive deformation prior to fracture. In contrast, the more tightly cross-linked ESO/CA network fails at lower elongation values.

Quantitative analysis of the mechanical parameters further confirms these differences. The ESO/CA system exhibits higher tensile strength (∼0.55 MPa vs ∼0.22 MPa), tensile stress at break (∼0.50 MPa vs ∼ 0.21 MPa), and elastic modulus (∼0.85 MPa vs ∼0.22 MPa) compared with ESO/SA. These results indicate that the network formed with CA provides a stiffer and stronger material, which can be attributed to its higher cross-link density and more efficient stress transfer through the polymer network.

Overall, the mechanical performance of these thermosets is strongly influenced by the chemical nature and functionality of the curing agents. The multifunctionality of citric acid promotes the formation of highly cross-linked and mechanically robust thermosets, whereas the lower cross-link density observed for the salicylic acid system results in materials with higher flexibility and elongation. Such differences may be advantageous for designing biobased epoxy materials with tailored mechanical properties for coatings, adhesives, and other polymeric applications. These findings are consistent with previous studies showing that increasing cross-link density in biobased epoxy networks generally enhances stiffness and strength while reducing ductility.^65–67^

In materials engineering, the balance between tensile strength and elongation is critical for determining the suitability of polymer networks for different applications. ESO/CA exhibited higher modulus and tensile strength, suggesting potential use in rigid materials or coatings where dimensional stability is required. In contrast, the higher elongation observed for ESO/SA indicates greater flexibility, which may be advantageous for applications requiring more compliant polymer networks. These differences highlight how the molecular structure and functionality of the curing agents influence the resulting network architecture and macroscopic mechanical behavior of the thermosets.

These results indicate that ESO/CA and ESO/SA networks present properties that may be interesting for the development of sustainable polymeric materials, including coatings and packaging-related applications, although further studies such as permeability and migration analyses would be required to confirm their suitability for food-contact systems.

### Main Achievements

- Demonstrated the effective cross-linking of epoxidized soybean oil (ESO) using carboxylic acids without the addition of catalysts, contributing to more sustainable thermoset resin systems;
- Established clear structure–property relationships relevant to food-contact materials, linking cross-link density and gel content to barrier potential, and identifying ESO/SA as a candidate for antimicrobial active packaging.
- Conducted advanced kinetic studies using isoconversional models (Friedman and Vyazovkin), revealing variable activation energy profiles throughout the curing process and highlighting the inadequacy of single- *E*_a_ models (Kissinger and Ozawa) for accurately describing these dynamic systems;
- Highlighted the use of curing agents with known regulatory status for potential food-contact applications, aligning material design with FDA and EU food safety requirements.

## Conclusions

A fully biobased epoxy system was successfully developed using epoxidized soybean oil (ESO) and carboxylic acids (citric, maleic, and salicylic acids) as curing agents. Among the acids investigated, maleic acid exhibited limited curing efficiency under the catalyst-free and moderate-temperature conditions employed in this study. Even after extended curing times, FTIR analysis revealed residual epoxy groups, and DSC thermograms lacked well-defined exothermic peaks, indicating incomplete epoxy conversion and restricted network formation. These findings are consistent with the low cross-link density observed for ESO/MA (∼0.3 mmol cm^–3^) and its high solvent uptake, which reflect limited cross-linking compared to the other systems.

In contrast, ESO/CA and ESO/SA formed well-developed, highly cross-linked networks, as confirmed by gel content and swelling measurements, with cross-link density values ranging from 1.7 to 2.1 mmol cm^–3^, in agreement with literature reports. The curing kinetics of ESO/CA and ESO/SA were successfully evaluated using the Friedman, Vyazovkin, Ozawa–Flynn–Wall, and Kissinger–Akahira–Sunose (KAS) models. Among these, the Friedman and Vyazovkin approaches provided the most reliable fits (*R* ^2^ > 0.99), whereas the Ozawa–Flynn–Wall and KAS models showed limitations due to their assumption of constant activation energy, making them less suitable for describing these complex curing systems. Activation energies reached up to 85 kJ mol^–1^ for ESO/CA (Friedman) and approximately 87 kJ mol^–1^ for ESO/SA (Vyazovkin). The slightly higher activation energy observed for ESO/SA may be associated with its aromatic structure and hydrogen-bonding capability, which contribute to increased network rigidity.

Mechanical testing further confirmed the structure–property relationships of the resulting thermosets. ESO/CA exhibited higher modulus and tensile strength, while ESO/SA showed greater elongation at break, reflecting differences in network architecture arising from the molecular structure and functionality of the curing agents.

Overall, this study provides a comprehensive investigation of the curing behavior and reaction kinetics of ESO-based thermosets prepared with different carboxylic acids. The combination of FTIR monitoring, DSC kinetic analysis, gel content, swelling measurements, and mechanical characterization allowed a detailed understanding of the network formation process and its influence on material properties. These findings demonstrate that ESO-based epoxy systems cured with suitable bioderived carboxylic acids can produce cross-linked thermosets with tunable properties, contributing to the development of sustainable biobased epoxy materials for coatings, adhesives, and related applications.

These findings contribute to the understanding of curing mechanisms in biobased epoxy systems and support the development of sustainable thermosets that may be further explored for coatings and packaging-related applications.

## Supplementary Material



## Notes

Coding and compositions of produced epoxy compounds; FTIR spectra curing compositions over time; Raw DSC thermograms at different heating rates; Thermogravimetric Analysis of Maleic Acid ate 10 °C/min; Linear regression of the Friedman Isoconversional, Kinssiger, OFW model at the rates indicated for the ESO/CA and ESO/SA compositions; Comparison between experimental values (lines) and theoretical values (dotted) of the relative degree of conversion (PDF)

## References

[1] Baroncini E. A., Yadav S. K., Palmese G. R., Stanzione J. F. (2016). Recent
advances in bio-based epoxy resins and bio-based epoxy curing agents. J. Appl. Polym. Sci..

[2] Khan F. M., Shah A. H., Wang S. (2022). A Comprehensive Review
on Epoxy Biocomposites Based on Natural Fibers and Bio-fillers: Challenges,
Recent Developments and Applications. Adv. Fiber
Mater..

[3] Shen Y., Le X., Wu Y., Chen T. (2024). Stimulus-responsive
polymer materials
toward multi-mode and multi-level information anti-counterfeiting:
recent advances and future challenges. Chem.
Soc. Rev..

[4] USDA-Grain World Mark . “USDA-grain: world markets and trade-2023,” Trade.

[5] Ramon E., Sguazzo C., Moreira P. M. G. P. (2018). A Review of
Recent Research on Bio-Based
Epoxy Systems for Engineering Applications and Potentialities in the
Aviation Sector. Aerospace.

[6] lal S. S., Sekar K. (2021). Recent Advances in
Bio-Based Sustainable Aliphatic and Aromatic Epoxy
Resins for Composite Applications. Key Eng.
Mater..

[7] Nepomuceno N. C., Barreto V., Wellen R. M. R. (2024). Effect of Dicarboxylic Acids’
Aliphatic Chain on the Curing of Epoxidized Soybean Oil (ESO) Resins. J. Polym. Environ..

[8] Barros A., Nepomuceno N., Nicácio P. (2024). Enhancement of Biobased
Epoxy Through the Curing and Thermal Stability Control with Carboxylic
Acids. J. Polym. Environ..

[9] Jiang Y., Li J., Li D. (2024). Bio-based hyperbranched epoxy resins: synthesis
and recycling. Chem. Soc. Rev..

[10] Pawar M., Kadam A., Yemul O., Thamke V., Kodam K. (2016). Biodegradable
bioepoxy resins based on epoxidized natural oil (cottonseed &
algae) cured with citric and tartaric acids through solution polymerization:
A renewable approach. Ind. Crops Prod..

[11] Malburet S., Di Mauro C., Noè C., Mija A., Sangermano M., Graillot A. (2020). Sustainable access
to fully biobased epoxidized vegetable
oil thermoset materials prepared by thermal or UV-cationic processes. RSC Adv..

[12] Galià M., de Espinosa L. M., Ronda J. C., Lligadas G., Cádiz V. (2010). Vegetable
oil-based thermosetting polymers. Eur. J. Lipid
Sci. Technol..

[13] Anusic A., Blößl Y., Oreski G., Resch-Fauster K. (2020). High-performance
thermoset with 100% bio-based carbon content. Polym. Degrad. Stab..

[14] Kadam A., Pawar M., Yemul O., Thamke V., Kodam K. (2015). Biodegradable
biobased epoxy resin from karanja oil. Polymer.

[15] Omonov T. S., Curtis J. M. (2014). Biobased epoxy resin
from canola oil. J. Appl. Polym. Sci..

[16] Ma S., Webster D. C. (2015). Naturally Occurring
Acids as Cross-Linkers To Yield
VOC-Free, High-Performance, Fully Bio-Based, Degradable Thermosets. Macromolecules.

[17] Verduyckt J., De Vos D. E. (2017). Highly selective
one-step dehydration, decarboxylation
and hydrogenation of citric acid to methylsuccinic acid. Chem. Sci..

[18] Kocaman S., Ahmetli G., Temiz M. (2024). Newly epoxy
resin synthesis from
citric acid and the effects of modified almond shell waste with different
natural acids on the creation of bio-based composites. Ind. Crops Prod..

[19] Moser B. R., Cermak S. C., Evangelista R. L. (2024). Fully biobased
epoxy resins from
ring opening polymerization of epoxidized pennycress (Thlaspi arvense
L.) oil with itaconic and citric acids. Ind.
Crops Prod..

[20] Peng J., Zhou C., Chen B. (2024). Rational design of waterborne
biobased epoxy methacrylates using citric acid and epoxy soybean oil
for UV-curable coatings. Ind. Crops Prod..

[21] Nepomuceno N. C., Bakkali-Hassani C., Wellen R., Caillol S., Negrell C. (2024). Fully bio-sourced
catalyst-free covalent adaptable networks from epoxidized soybean
oil and L-tartaric acid. Eur. Polym. J..

[22] Thiyagarajan S., Franciolus D., Bisselink R. J. M., Ewing T. A., Boeriu C. G., van Haveren J. (2020). Selective
Production of Maleic Acid from Furfural via
a Cascade Approach Combining Photochemistry and Electro- or Biochemistry. ACS Sustainable Chem. Eng..

[23] Faggio N., Marotta A., Ambrogi V., Cerruti P., Gentile G. (2023). Fully bio-based
furan/maleic anhydride epoxy resin with enhanced adhesive properties. J. Mater. Sci..

[24] Sulthan R., Reghunadhan A., Sreenath R., Shankar B., Sambhudevan S. (2024). Preparation,
physicochemical analyses, and comparative evaluation studies of deep
eutectic solvents (DES) from non-conventional precursor materials
applied as catalytic curing agents for epoxy resin. J. Mol. Liq..

[25] Nepomuceno N. C., Fook M. V. L., Ries A., Mija A., Wellen R. M. R. (2023). Bio-Based
Epoxy Resins of Epoxidized Soybean oil Cured with Salicylic acid Loaded
with Chitosan: Evaluation of Physical–Chemical Properties. J. Polym. Environ..

[26] Métraux J.-P. (2002). Recent
breakthroughs in the study of salicylic acid biosynthesis. Trends Plant Sci..

[27] Lo’ay A. A., Taher M. A. (2018). Effectiveness salicylic
acid blending in chitosan/PVP
biopolymer coating on antioxidant enzyme activities under low storage
temperature stress of ‘Banati’ guava fruit. Sci. Hortic..

[28] Molamohammadi H., Pakkish Z., Akhavan H.-R., Saffari V. R. (2020). Effect
of Salicylic
Acid Incorporated Chitosan Coating on Shelf Life Extension of Fresh
In-Hull Pistachio Fruit. Food Bioprocess Technol..

[29] Li J., Zhao H., Sui G. (2022). Renewable green reactive diluent
for bisphenol a epoxy resin system: curing kinetics and properties. RSC Adv..

[30] Barreto J., Soares N., Souza M. (2024). Curing and degradation
kinetics of crosslinked epoxidized soybean oil with isosorbide-based
curing agent. J. Environ. Chem. Eng..

[31] Bu M., Zhang X., Zhou T., Lei C. (2022). Fully bio-based epoxy
resins derived from magnolol and varying furan amines: Cure kinetics,
superior mechanical and thermal properties. Eur. Polym. J..

[32] Barreto J. V. M., de Albuquerque A. K. C., Jacques N. G., Wellen R. M. R. (2023). On the
curing and degradation of bisphenol A diglycidyl ether and epoxidized
soybean oil compounds cured with itaconic and succinic acids. J. Appl. Polym. Sci..

[33] Flory P. J., Rehner J. (1943). Statistical Mechanics
of Cross-Linked Polymer Networks
I. Rubberlike Elasticity. J. Chem. Phys..

[34] Friedman H.
L. (1964). Kinetics
of thermal degradation of char-forming plastics from thermogravimetry.
Application to a phenolic plastic. J. Polym.
Sci., Part C: Polym. Symp..

[35] Vyazovkin S. (2001). Modification
of the integral isoconversional method to account for variation in
the activation energy. J. Comput. Chem..

[36] Vyazovkin S. (2006). Model-free
kinetics. J. Therm. Anal. Calorim..

[37] Doyle C. D. (1962). Estimating
isothermal life from thermogravimetric data. J. Appl. Polym. Sci..

[38] Hiba, R. ; Emna, B. B. ; Mohamed Hachemi, C. Kinetic Study of Waste Tires by Thermogravimetric Analysis (TGA): Kissinger–Akahira–Sunose (KAS) Method. In Environmental Science and Engineering; Springer Nature, 2021; pp 317–321 10.1007/978-3-030-51210-1_52.

[39] Coats A. W., Redfern J. P. (1964). Kinetic Parameters from Thermogravimetric
Data. Nature.

[40] Tang, Q. ; Chen, Y. ; Gao, H. Bio-Based Epoxy Resin from Epoxidized Soybean Oil. In Soybean - Biomass, Yield and Productivity; IntechOpen, 2019 10.5772/intechopen.81544.

[41] Silverstein, R. M. ; Webster, F. X. ; Kiemle, D. Spectrometric Identification of Organic Compounds 7th ed.; 2005.

[42] Stemmelen M., Pessel F., Lapinte V., Caillol S., Habas J.-P., Robin J.-J. (2011). A fully
biobased epoxy resin from vegetable oils: From
the synthesis of the precursors by thiol-ene reaction to the study
of the final material. J. Polym. Sci. A: Polym.
Chem..

[43] Falco G., Sbirrazzuoli N., Mija A. (2019). Biomass derived epoxy systems: From
reactivity to final properties. Mater. Today
Commun..

[44] Kim H., Kim J., Kim D. (2021). Enhancement of Gel Strength of Itaconic Acid-Based
Superabsorbent Polymer Composites Using Oxidized Starch. Polymers.

[45] Pavia, D. L. ; Lampman, G. M. ; Kriz, G. S. ; Vyvyan, J. R. Introduction to Spectroscopy; Cengage Learning, 2013; Vol. 5.

[46] Ding C., Shuttleworth P. S., Makin S., Clark J. H., Matharu A. S. (2015). New insights
into the curing of epoxidized linseed oil with dicarboxylic acids. Green Chem..

[47] Ding C., Matharu A. S. (2014). Recent Developments
on Biobased Curing Agents: A Review
of Their Preparation and Use. ACS Sustainable
Chem. Eng..

[48] Lascano D., Lerma-Canto A., Fombuena V., Balart R., Montanes N., Quiles-Carrillo L. (2021). Kinetic Analysis of the Curing Process of Biobased
Epoxy Resin from Epoxidized Linseed Oil by Dynamic Differential Scanning
Calorimetry. Polymers.

[49] Noè C., Hakkarainen M., Sangermano M. (2021). Cationic UV-Curing of Epoxidized
Biobased Resins. Polymers.

[50] Gobin M., Loulergue P., Audic J.-L., Lemiègre L. (2015). Synthesis
and characterisation of bio-based polyester materials from vegetable
oil and short to long chain dicarboxylic acids. Ind. Crops Prod..

[51] Frias C. F., Serra A. C., Ramalho A., Coelho J. F. J., Fonseca A. C. (2017). Preparation
of fully biobased epoxy resins from soybean oil based amine hardeners. Ind. Crops Prod..

[52] Tran T.-N., Mauro C. Di., Graillot A., Mija A. (2020). Chemical Reactivity
and the Influence of Initiators on the Epoxidized Vegetable Oil/Dicarboxylic
Acid System. Macromolecules.

[53] Zeng R.-T., Wu Y., Li Y.-D., Wang M., Zeng J.-B. (2017). Curing behavior
of epoxidized soybean oil with biobased dicarboxylic acids. Polym. Test..

[54] Dhar S., Pathak M., Shukla P. R. (2020). Transformation
of India’s
steel and cement industry in a sustainable 1.5 °C world. Energy Policy.

[55] Tan S. G., Chow W. S. (2011). Curing Characteristics
and Thermal Properties of Epoxidized
Soybean Oil Based Thermosetting Resin. J. Am.
Oil Chem. Soc..

[56] Li J., Ju B., Zhang S. (2023). Catalyst-free, sustainable epoxy
vitrimers from epoxidized
soybean oil and natural sugar alcohols. Ind.
Crops Prod..

[57] Khundamri N., Aouf C., Fulcrand H., Dubreucq E., Tanrattanakul V. (2019). Bio-based
flexible epoxy foam synthesized from epoxidized soybean oil and epoxidized
mangosteen tannin. Ind. Crops Prod..

[58] Allasia M., Estevez V. G., Chesta A. A. (2021). New insights into the
properties of alkali-degradable thermosets based on epoxidized soy
oil and plant-derived dicarboxylic acids. Polymer.

[59] Li L., Zeng Z., Zou H., Liang M. (2015). Curing characteristics
of an epoxy resin in the presence of functional graphite oxide with
amine-rich surface. Thermochim. Acta.

[60] Bratasyuk N. A., Zuev V. V. (2020). The study of the
curing mechanism, kinetic and mechanical
performance of polyurethane/epoxy composites using aliphatic and aromatic
amines as curing agents. Thermochim. Acta.

[61] Achilias D. S., Karabela M. M., Varkopoulou E. A., Sideridou I. D. (2012). Cure Kinetics
Study of Two Epoxy Systems with Fourier Tranform Infrared Spectroscopy
(FTIR) and Differential Scanning Calorimetry (DSC). J. Macromol. Sci., Part A.

[62] Šimon P. (2004). Isoconversional
methods. J. Therm. Anal. Calorim..

[63] Zhou T., Gu M., Jin Y., Wang J. (2005). Studying on the curing kinetics of
a DGEBA/EMI-2,4/nano-sized carborundum system with two curing kinetic
methods. Polymer.

[64] Ferdosian F., Zhang Y., Yuan Z., Anderson M., Xu C. C. (2016). Curing
kinetics and mechanical properties of bio-based epoxy composites comprising
lignin-based epoxy resins. Eur. Polym. J..

[65] Roudsari G. M., Mohanty A. K., Misra M. (2014). Study of the
Curing Kinetics of Epoxy
Resins with Biobased Hardener and Epoxidized Soybean Oil. ACS Sustainable Chem. Eng..

[66] Criado J. M., Sánchez-Jiménez P. E., Pérez-Maqueda L. A. (2008). Critical
study of the isoconversional methods of kinetic analysis. J. Therm. Anal. Calorim..

[67] Zhang C., Ma S., Wang Y., Zhu J. (2019). Recent advances in bio-based epoxy
resins: Review of synthesis and performance. J. Polym. Environ..

