# Supplementary material for: Carboxylic Acid-Cured Epoxidised Soybean Oil under Catalyst-Free Conditions: Kinetics and Sustainable Packaging Potential
Source: ACS Omega. 2026 Aug 18;11(36):53745–59. doi: 10.1021/acsomega.5c11969 (PMC13584165; doi:10.1021/acsomega.5c11969)
Supplement: Supplementary file 1 [file ao5c11969_si_001.pdf]

Carboxylic Acid-Cured Epoxidised Soybean Oil under Catalyst-Free Conditions: Kinetics and Sustainable Packaging Potential

Ana Barros[1], Neymara Nepomuceno[1], Andreas Ries[2], Carlos Luna[1], Marcus Fook[1], Edcleide Araújo[1], Renate Wellen[1,3]

[1] Academic Unit of Materials Engineering, Federal University of Campina Grande, Av. Aprígio Veloso, 882 - Bodocongó, 58429-900 Campina Grande, Paraíba, Brazil

[2] Polytechnic Faculty, National University of Asunción, 111421 San Lorenzo, Paraguay

[3] Department of Materials Engineering, Federal University of Paraíba, Cidade Universitária, 58051-900 João Pessoa, PB, Brazil

Correspondence should be addressed to: bsbarros01@gmail.com

SUPPLEMENTARY MATERIAL

Table S1 – Coding and compositions of produced epoxy compounds.

Compound	Citric acid - CA (g)	Maleic acid - MA (g)	Salicylic acid - SA (g)	Epoxidized soybean oil - ESO (g)	Total mass (g)
**ESO/CA**	1.3012	-	-	5.826	7.1272
**ESO/MA**	-	1.1270	-	5.567	6.6940
**ESO/SA**	-	-	1.2974	5.387	6.6844

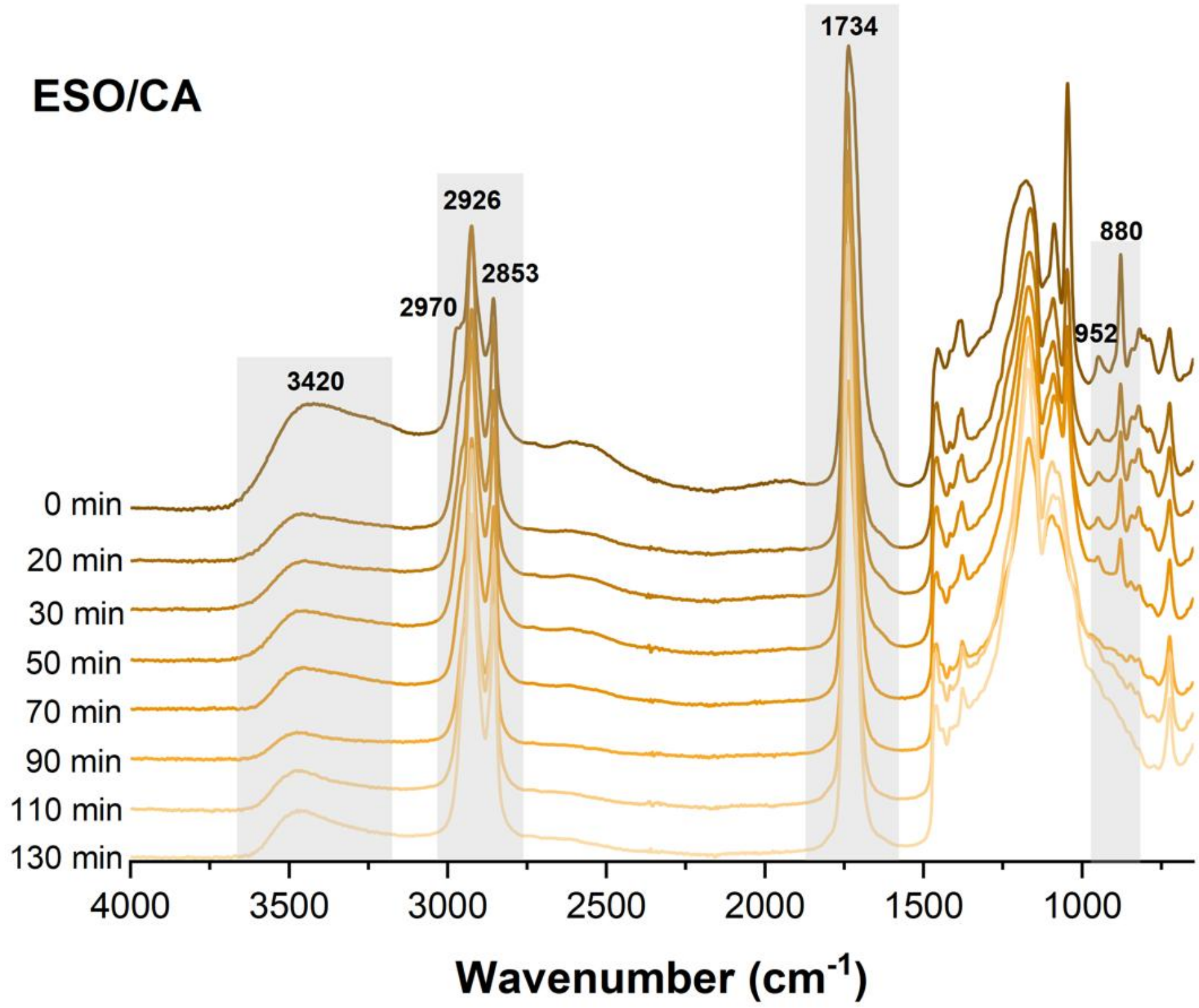

Figure S1: FTIR spectrums for ESO/CA curing compositions over time.

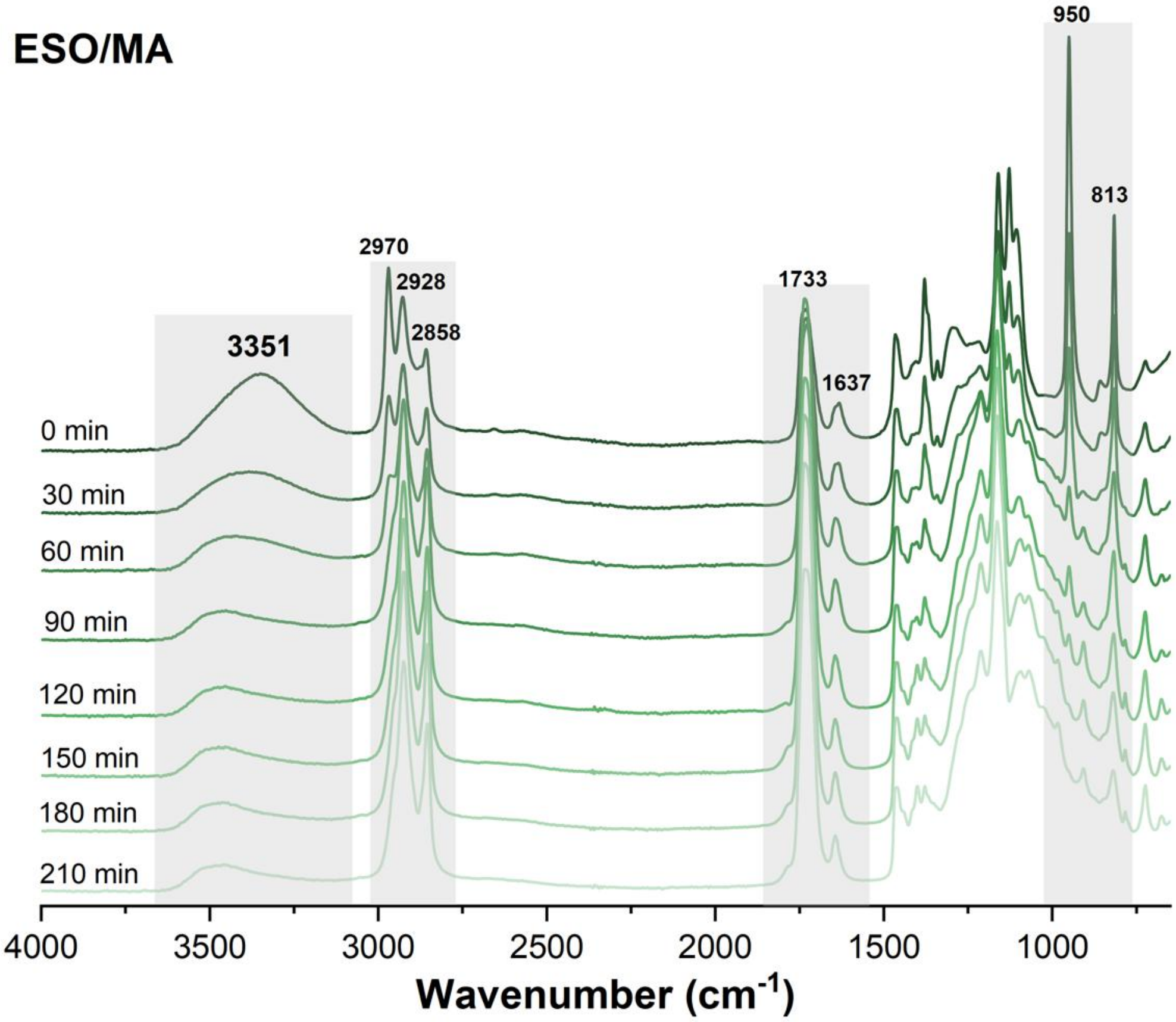

Figure S2: FTIR spectrums for ESO/MA curing compositions over time.

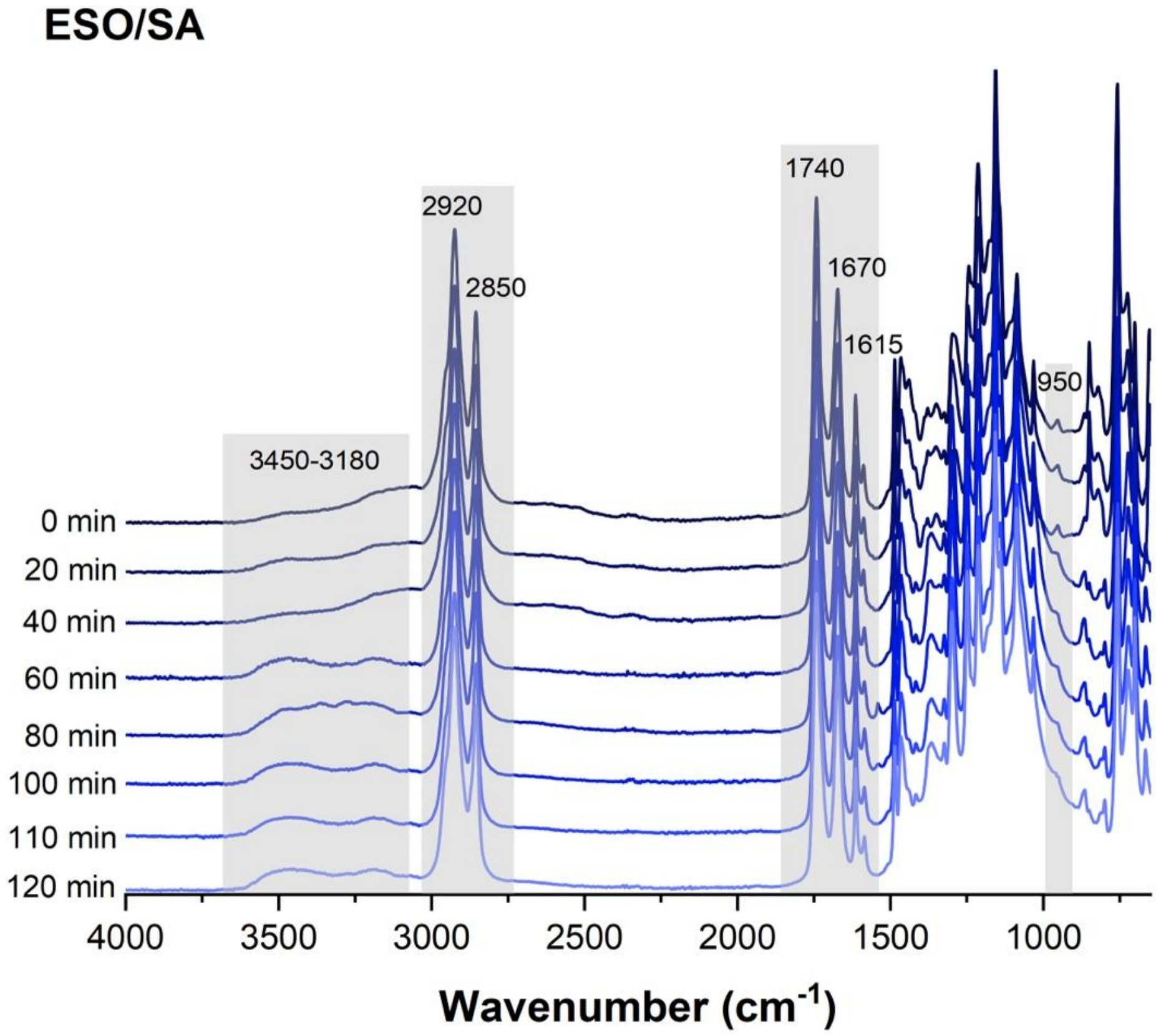

Figure S3: FTIR spectrums for ESO/MA curing compositions over time.

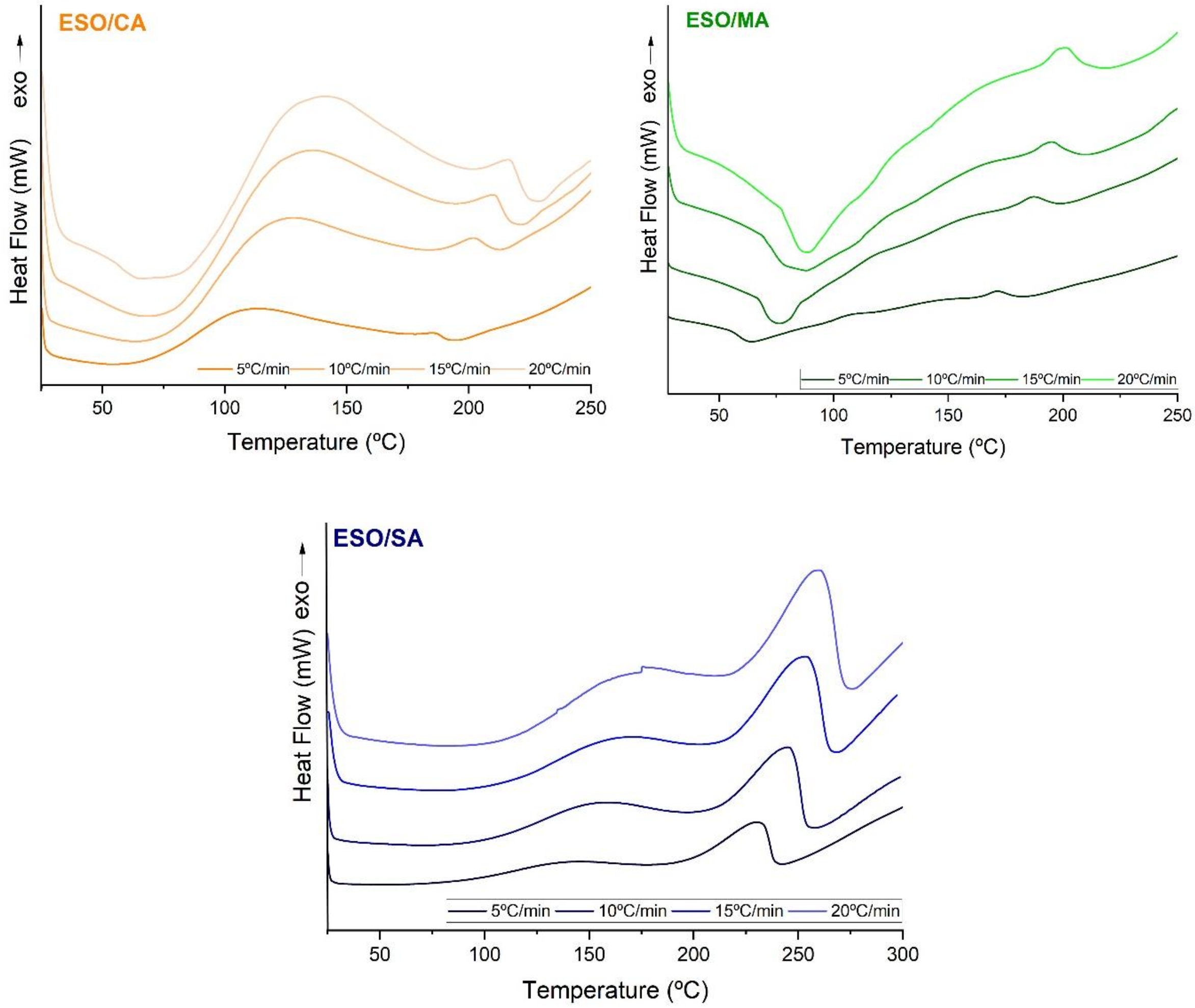

Figure S4: DSC curves for ESO/CA(a), ESO/MA(b) and ESO/SA(c) at different heating rates (5°C/min, 10°C/min, 15°C /min and 20°C/min).

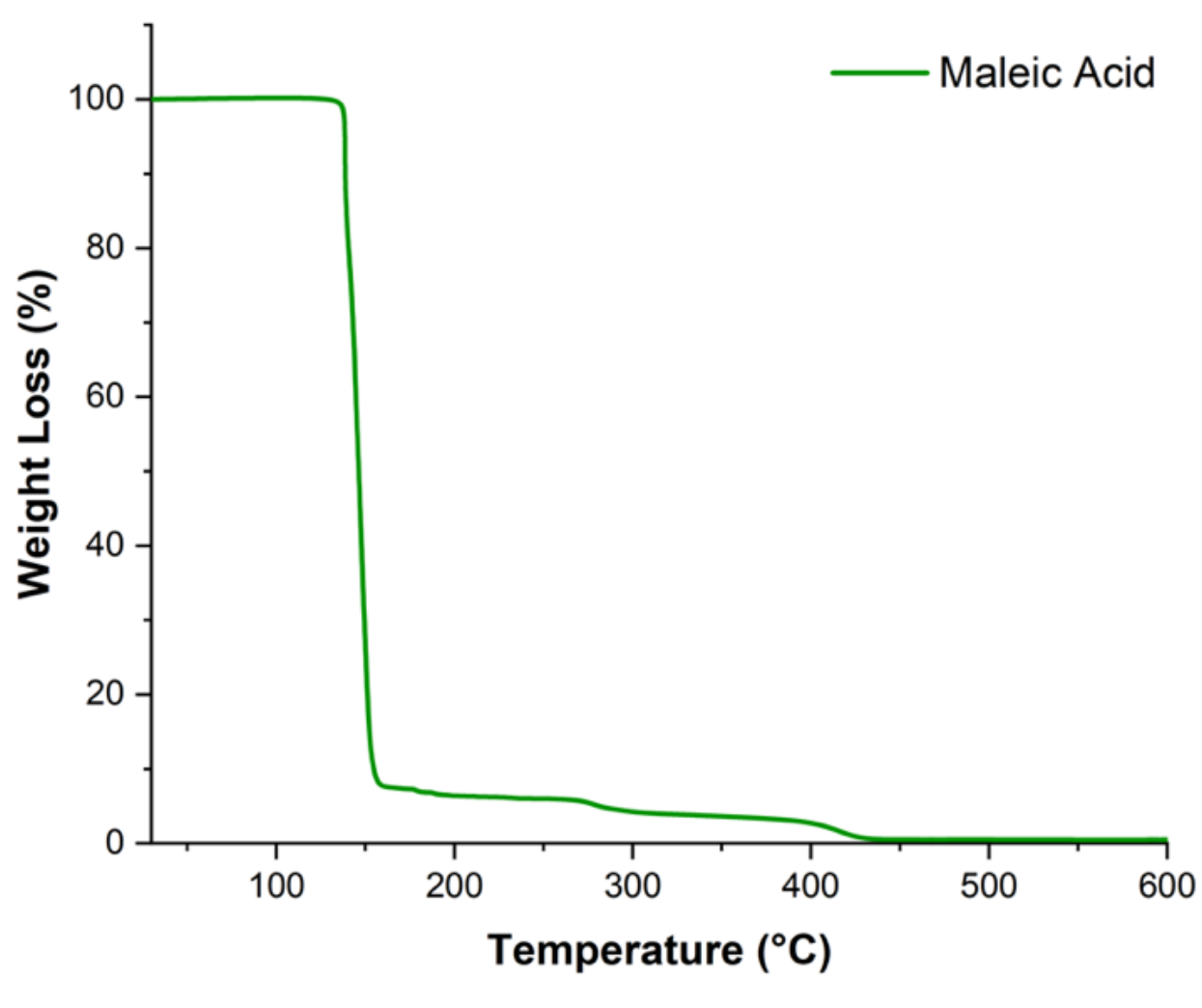

Figure S5: Thermogravimetric Analysis of Maleic Acid ate 10°C/min.

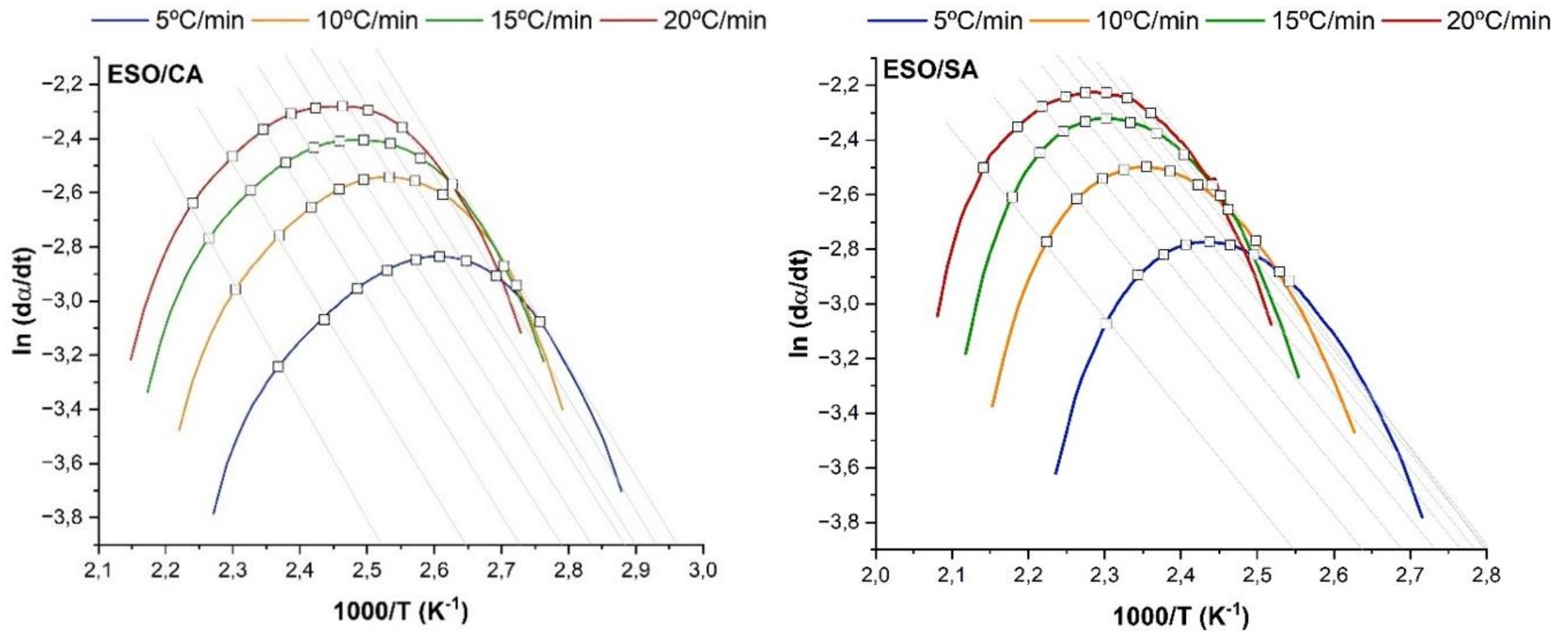

Figure S6. Linear regression of the Friedman Isoconversional model ln (dα/dt) versus 1000/Tp at the rates indicated for the ESO/CA and ESO/SA compositions.

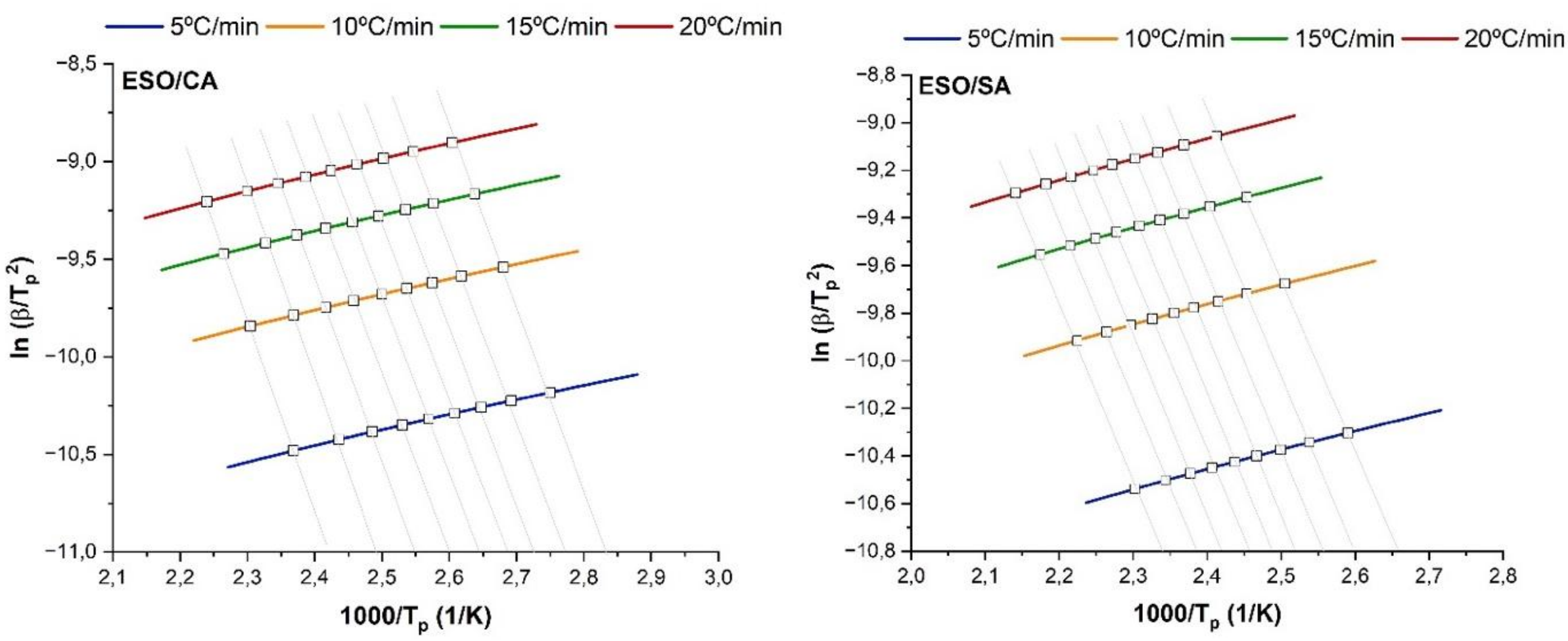

Figure S7. Linear regression of the Kinssiger model on the rates indicated for the ESO/CA and ESO/SA compositions.

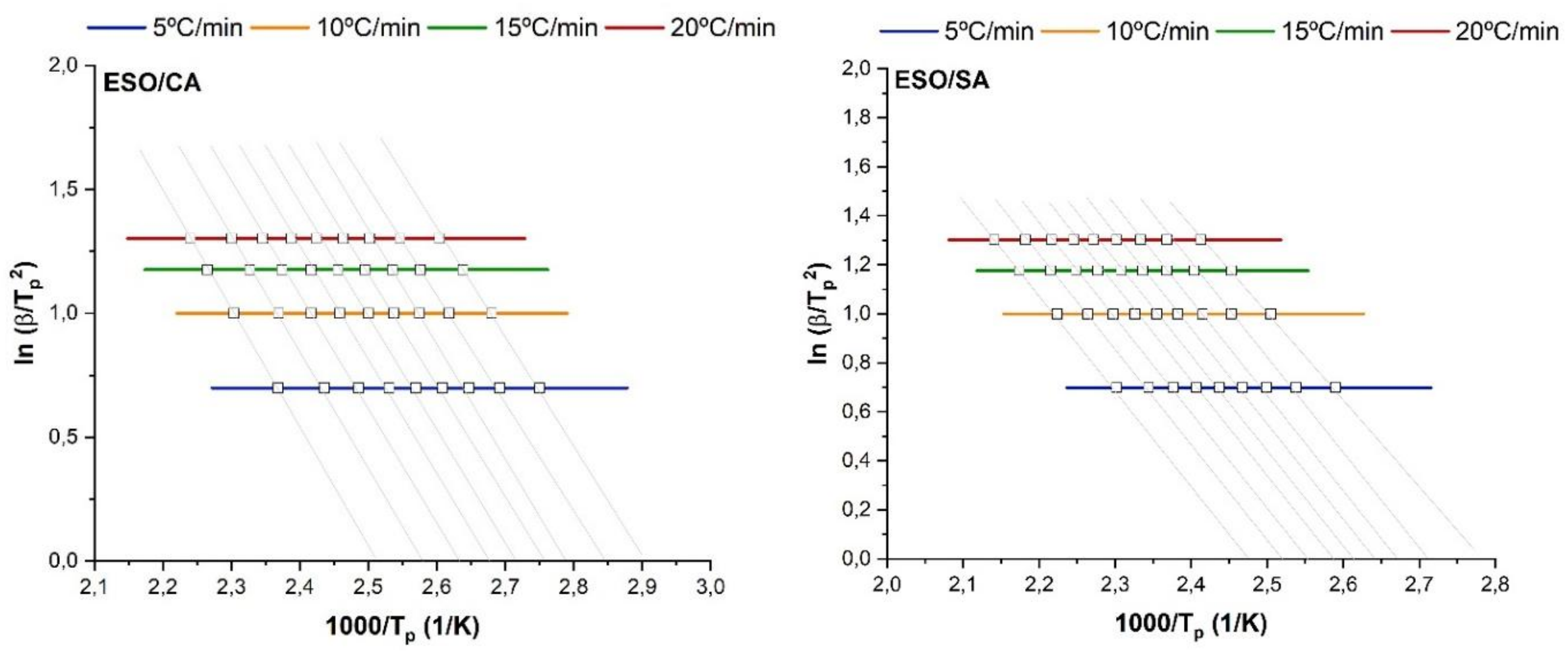

Figure S8. Linear regression of the OFW model for the ESO/CA and ESO/SA compositions.

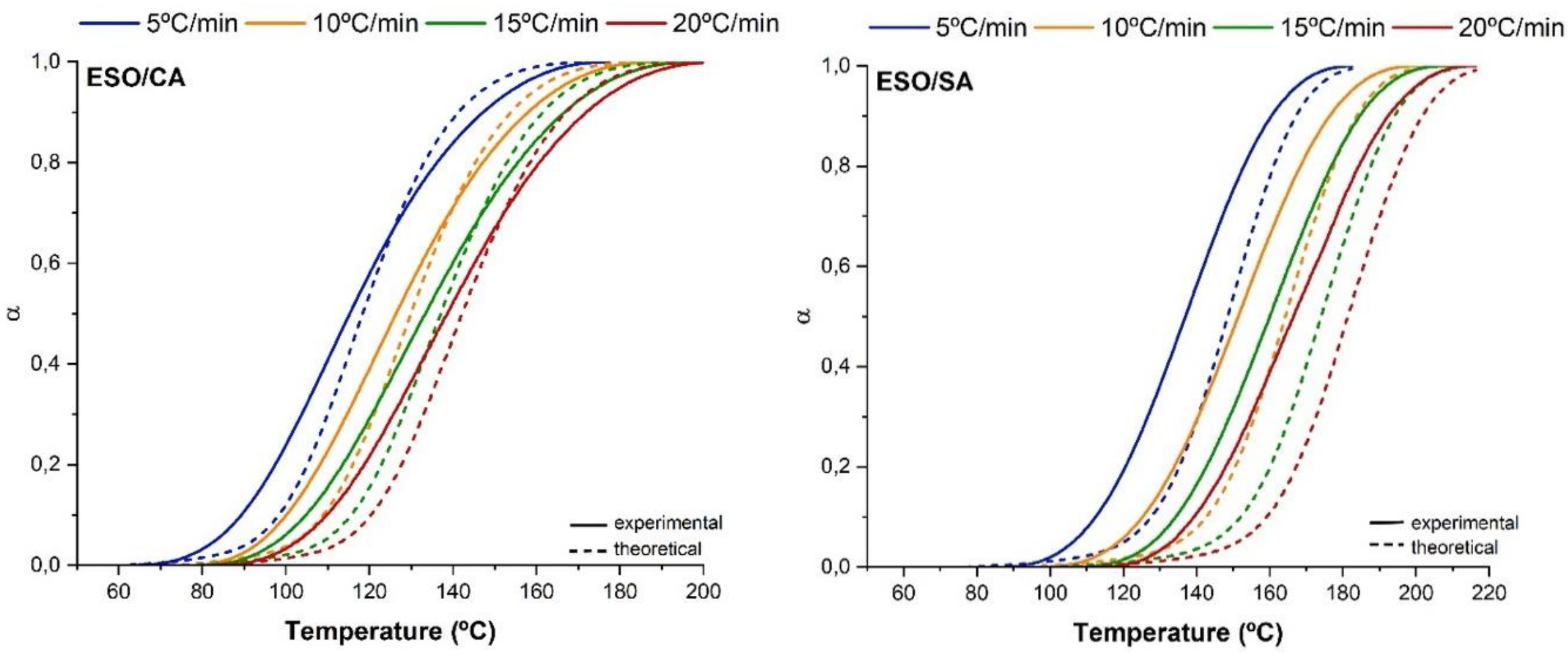

Figure S9. Comparison between experimental values (lines) and theoretical values (dotted) of the relative degree of conversion as a function of temperature estimated by the Kissinger model for ESO/CA and ESO/SA at the indicated rates.

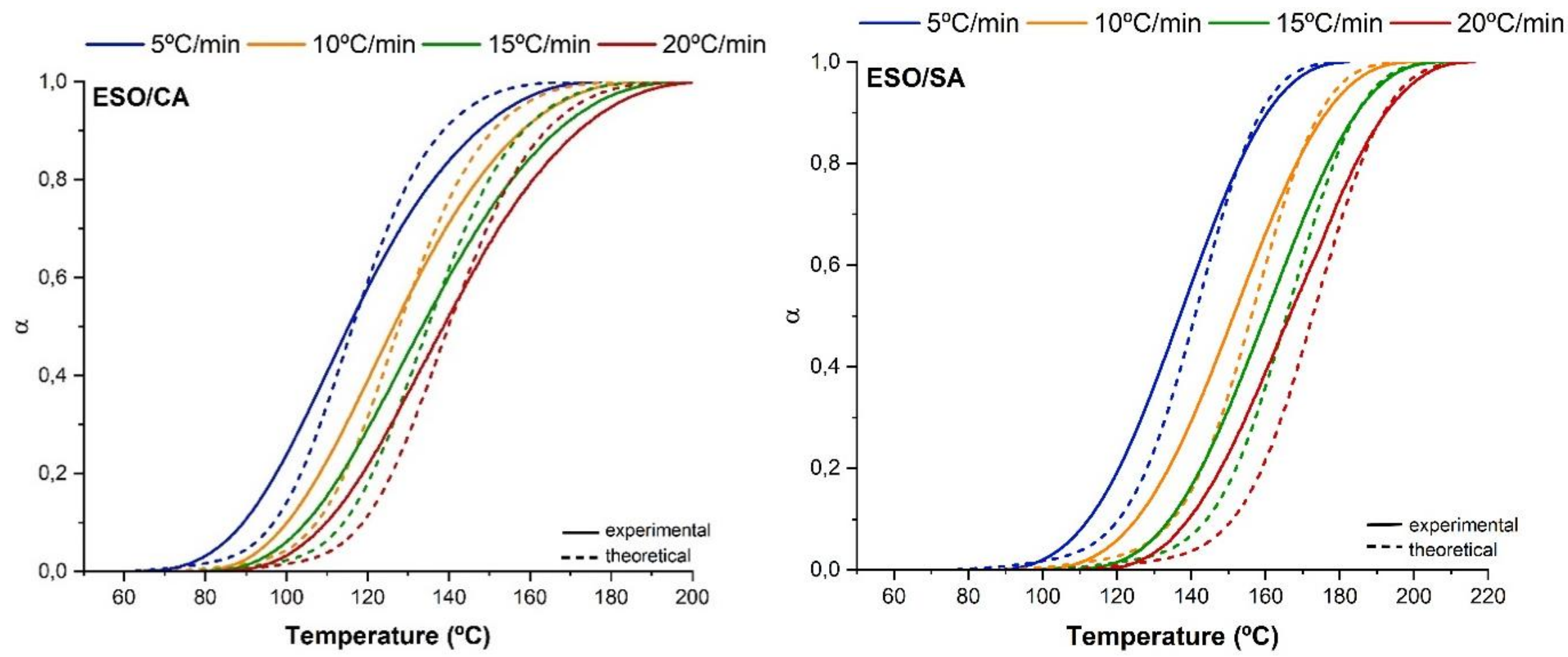

Figure S10. Comparison between experimental values (lines) and theoretical values (dotted) of the relative degree of conversion as a function of temperature estimated by the Ozawa model for ESO/CA and ESO/SA at the indicated rates.
